# Supplementary material for: JMJD3 acts in tandem with KLF4 to facilitate reprogramming to pluripotency
Source: Nat Commun. 2020 Oct 8;11:5061. doi: 10.1038/s41467-020-18900-z (PMC7545202; doi:10.1038/s41467-020-18900-z)
Supplement: Supplementary file 1 — Supplementary Information [file 41467_2020_18900_MOESM1_ESM.pdf]

Supplementary Information

JMJD3 acts in tandem with KLF4 to facilitate reprogramming to pluripotency

Huang et al.

Supplementary Figure 1

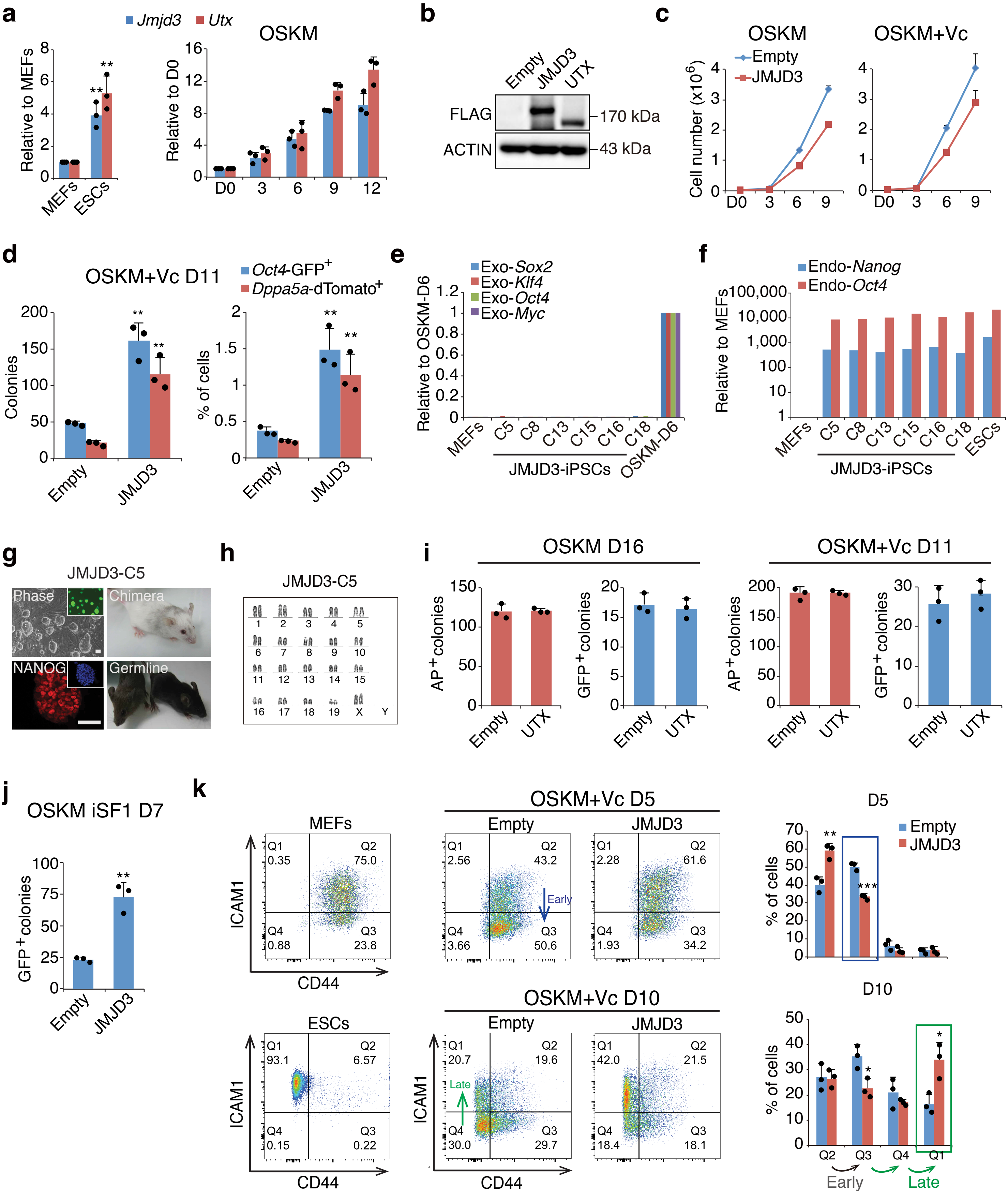

Supplementary Figure 1 JMJD3 plays both negative and positive roles in reprogramming.

(**a**) RT-qPCR for *Jmjd3* and *Utx* in P2 MEFs, ESCs (OG2 ESCs) and an OSKM-reprogramming time course.
(**b**) Western blotting for FLAG showing exogenous FLAG-tagged JMJD3 or UTX expression in OSKM-reprogrammed P2 MEFs. ACTIN serves as the loading control.
(**c**) Growth curves of OSKM-reprogrammed MEFs with Empty or JMJD3 in medium with or without Vc at the indicated time points.
(**d**) Number of *Oct4*-GFP^+ and *Dppa5a*-$dTomato^+$ colonies (left panel) and percentage of positive cells for each, measured by flow cytometry (right panel) in OSKM-reprogrammed dual-reporter P2 MEFs with Empty or JMJD3.
(**e**,**f**) Silencing of the OSKM transgenes (**e**) and activation of endogenous pluripotency genes (*Oct4* and *Nanog*; **f**) in iPSC clones produced with exogenous JMJD3. MEFs were used as negative control and OSKM-D6 (cells at day 6 of OSKM reprogramming; in **e**) and ESCs (OG2 ESCs; in **f**) as positive controls. Data were plotted from one experiment with three technical replicates.
(**g**,**h**) Phase contrast, *Oct4*-GFP fluorescence, NANOG immunofluorescence, chimeric mice, germline transmission (**g**) and karyotype analysis (**h**) of JMJD3-C5 iPSCs. Scale bars, 50 µm.
(**i**) Number of AP^+ and GFP^+ colonies in OSKM-reprogrammed P2 MEFs with Empty or UTX at day 16 (without Vc, left panel) and day 11 (with Vc, right panel).
(**j**) Number of GFP^+ colonies at day 7 in OSKM-reprogrammed P2 MEFs with Empty or JMJD3 in iSF1 medium.
(**k**) Flow cytometry analysis of cell surface markers CD44 and ICAM1 in OSKM-reprogrammed P2 MEFs with Empty or JMJD3 at days 5 (early) and 10 (late), with MEFs and ESCs (OG2 ESCs) as controls.
Error bars represent the s.e.m.. Data are presented as mean ± s.e.m. from n=3 (**a**,**c**,**d**,**i**-**k**) biologically independent experiments. Statistical analyses were performed using a two-tailed unpaired Student's *t*-test ($*P < 0.05$; $**P < 0.01$; $***P < 0.001$). *P* values: 0.0024, 0.0030 (**a**); 0.0013, 0.0022, 0.0030, 0.0051 (**d**); 0.0017 (**j**); 0.0051, 0.0004, 0.0207, 0.0184 (**k**). Experiments were repeated independently three times (**b**) with similar results or once (**g**). Source data are provided as a Source Data file.

Supplementary Figure 2

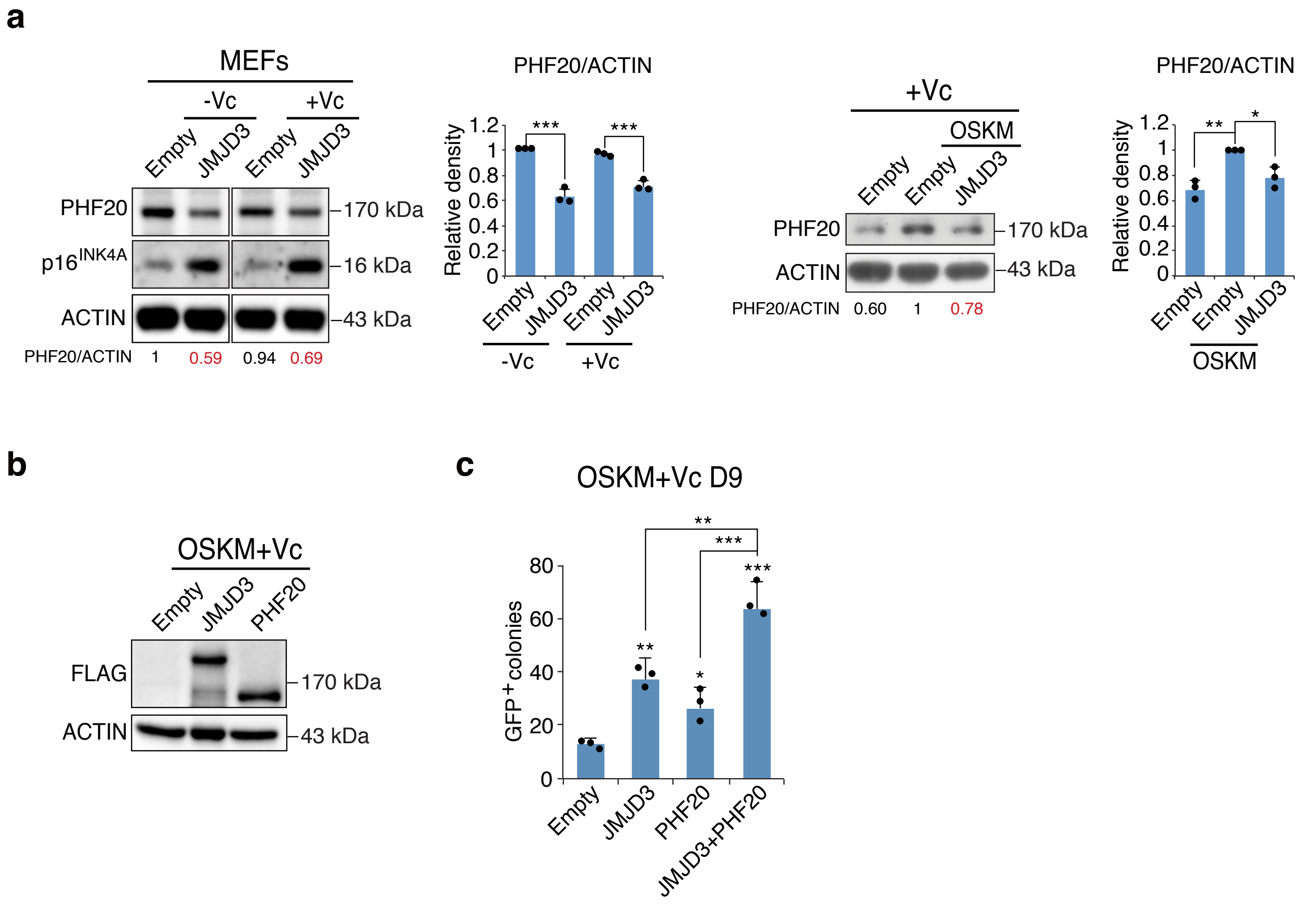

Supplementary Figure 2 The effect of JMJD3 on PHF20 during reprogramming.

(**a**) Western blotting for PHF20 and p16^{INK4A} in P2 MEFs transduced with Empty or JMJD3 in MEF medium with or without Vc (left panels), and for PHF20 in MEFs transduced with Empty or OSKM with or without JMJD3 in reprogramming medium with Vc (right panels). The quantification and statistics for PHF20 (normalized to ACTIN) in three independent experiments is shown.

(**b**) Western blotting for FLAG-tagged JMJD3 and PHF20 using FLAG antibody.

(**c**) Number of GFP$^+$ colonies in OSKM-reprogrammed P2 MEFs with the indicated genes.

All error bars throughout the figure represent the standard error of mean (s.e.m.). Data are presented as mean ± s.e.m. from n=3 (**a,c**) biologically independent experiments. Statistical analyses were performed using a two-tailed unpaired Student's t-test (**a**), or one-way ANOVA followed by a Holm-Sidak multiple comparison test (**c**) ($^*P < 0.05$; $^{**}P < 0.01$; $^{***}P < 0.001$). P values: 0.0005, 0.0007, 0.0018, 0.0102 (**a**); 0.0022, 0.0392, 0.0001, 0.0013 (JMJD3+PHF20 vs JMJD3), 0.0002 (JMJD3+PHF20 vs PHF20) (**c**). Experiments were performed once (**b**). Source data are provided as a Source Data file.

Supplementary Figure 3

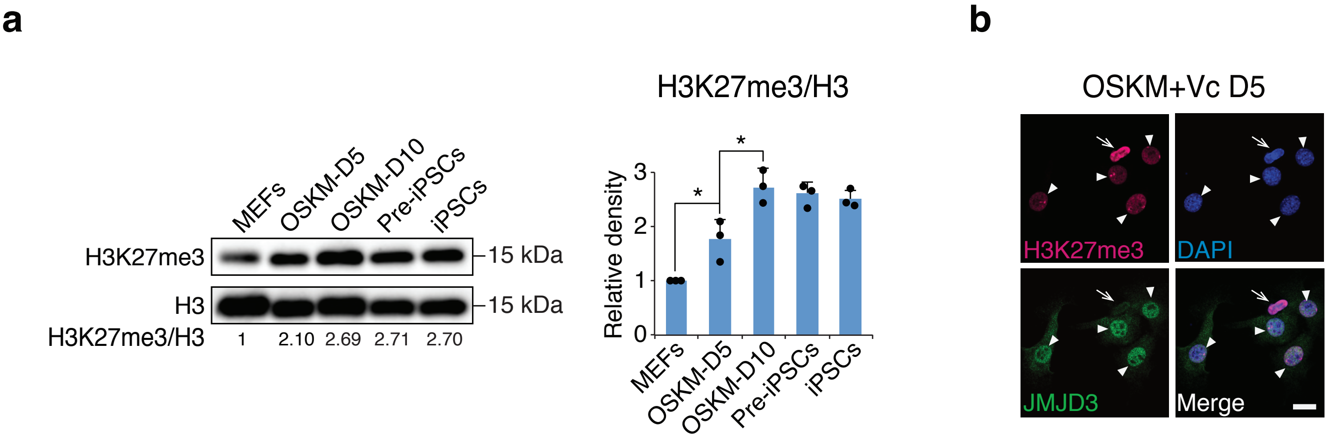

Supplementary Figure 3 JMJD3 globally reduces H3K27me3 during reprogramming.

(**a**) Western blotting, quantification and statistics for H3K27me3 in MEFs, OSKM reprogramming MEFs (day 5 and day 10), pre-iPSCs and OSK-iPSCs.
(**b**) Immunofluorescence showing the effect of exogenous JMJD3 compared to Empty on H3K27me3 levels in OSKM-reprogrammed P2 MEFs. Scale bar, 20 μm. Arrows mark cells without exogenous JMJD3 and arrowheads mark cells with exogenous JMJD3.
Error bars represent the s.e.m.. Data are presented as mean ± s.e.m. from n=3 (**a**) biologically independent experiments. Statistical analyses were performed using a two-tailed unpaired Student's t-test ($*P < 0.05$). P values: 0.0225, 0.0328 (**a**). Experiments were repeated independently three times (**b**) with similar results. Source data are provided as a Source Data file.

Supplementary Figure 4

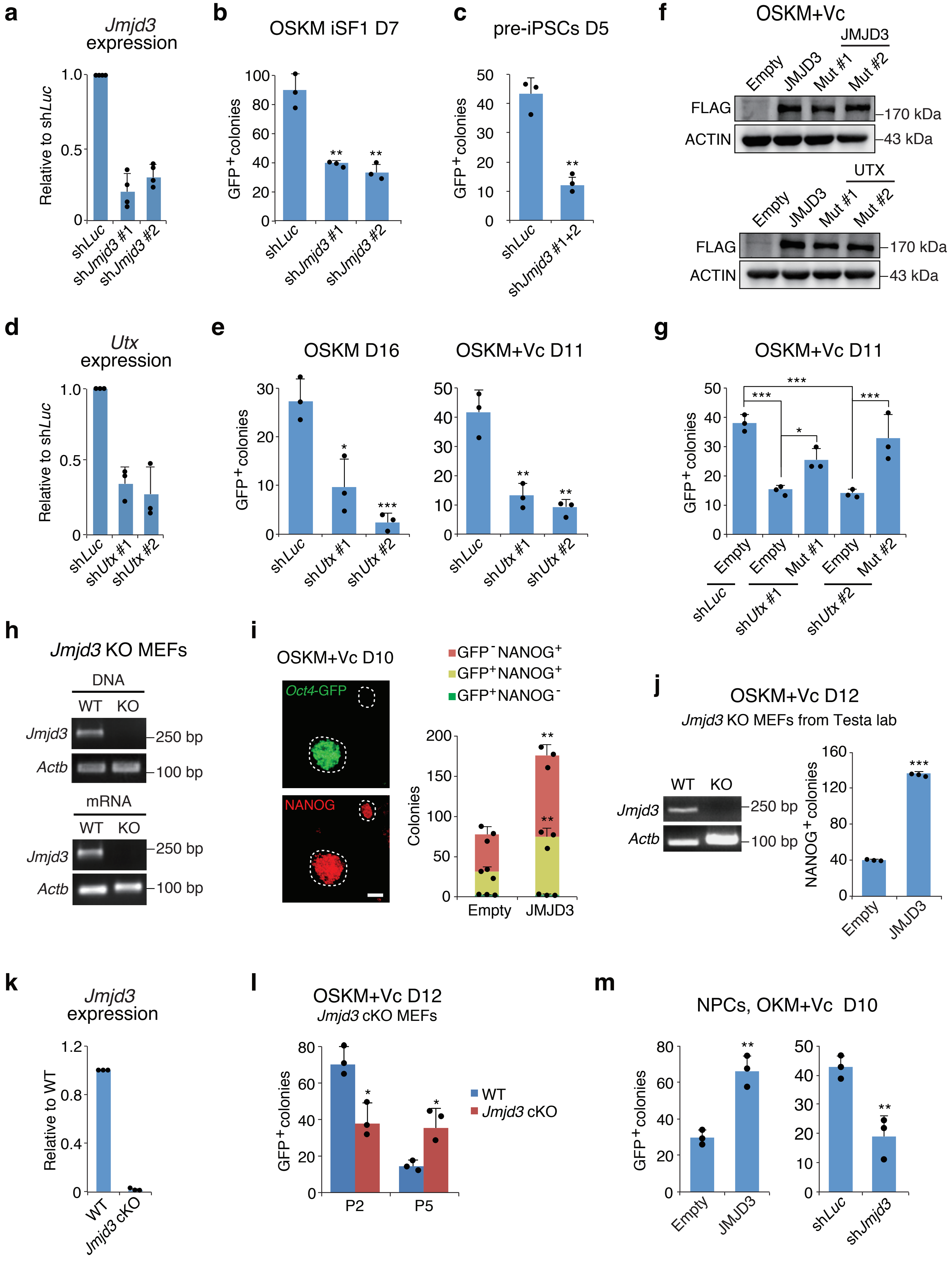

Supplementary Figure 4 Endogenous JMJD3 is important for efficient reprogramming.

(**a**) RT-qPCR analysis showing the knockdown efficiency of sh*Jmjd3*.

(**b**) Number of GFP$^+$ colonies in OSKM-reprogrammed P2 MEFs with sh*Luc* or sh*Jmjd3* in iSF1 medium.

(**c**) Number of GFP$^+$ colonies in pre-iPSCs with sh*Luc* or sh*Jmjd3*.

(**d**) RT-qPCR analysis showing the knockdown efficiency of sh*Utx*.

(**e**) Number of GFP$^+$ colonies at days 11 (with Vc) and 16 (without Vc) in OSKM-reprogrammed P2 MEFs with sh*Luc* or sh*Utx*.

(**f**) Western blotting for FLAG to show exogenous FLAG-tagged JMJD3- (upper panel) and UTX-rescue mutants (lower panel) in OSKM-reprogrammed P2 MEFs.

(**g**) Number of GFP$^+$ colonies in OSKM-reprogrammed P2 MEFs with sh*Luc* or sh*Utx*, together with the indicated UTX-rescue mutants or Empty.

(**h**) PCR with genomic DNA and RT-PCR confirmation for *Jmjd3* KO MEFs.

(**i**) Representative images of NANOG immunostaining for OG2 MEFs reprogrammed with OSKM (left panel) and colony numbers for each marker of the same OSKM-reprogramming experiment with Empty or JMJD3 (right panel). Scale bar, 100 µm.

(**j**) RT-PCR confirmation (left panel) and reprogramming efficiency (right panel) of a second source of *Jmjd3* KO MEFs (P2) with Empty or JMJD3.

(**k**) RT-qPCR for *Jmjd3* in P2 WT and *Jmjd3* cKO MEFs.

(**l**) Number of GFP$^+$ colonies in P2 and P5 WT and *Jmjd3* cKO MEFs reprogrammed with OSKM.

(**m**) Number of GFP$^+$ colonies in NPCs reprogrammed with OKM (OCT4, KLF4 and c-MYC) and JMJD3 OE or knockdown.

Error bars represent the s.e.m.. Data are presented as mean ± s.e.m. from n=3 (**a**-**e**,**g**,**i**-**m**) biologically independent experiments. Statistical analyses were performed using a two-tailed unpaired Student's t-test (**a**-**e**,**i**-**m**), or one-way ANOVA followed by a Holm-Sidak multiple comparison test (**g**) (*$P < 0.05$; **$P < 0.01$; ***$P < 0.001$). P values: 0.0046, 0.0011 (**b**); 0.0011 (**c**); 0.0135, 0.0009, 0.0048, 0.0023 (**e**); 0.0002 (sh*Utx* #1+Empty vs sh*Luc*+Empty), 0.0159 (sh*Utx* #1+Mut #1 vs sh*Utx* #1+Empty), 0.0002 (sh*Utx* #2+Empty vs sh*Luc*+Empty), 0.0007 (sh*Utx* #2+Mut #2 vs sh*Utx* #2+Empty) (**g**); 0.0043,0.0038 (**i**); 7.38×10^{-8} (**j**); 0.0199, 0.0311 (**l**); 0.0027, 0.0072 (**m**). Experiments were repeated independently twice (**f**) with similar results or once (**h**). Source data are provided as a Source Data file.

Supplementary Figure 5

a

OSKM+Vc

JMJD3 OE	Up	Down
Early (D5)	320 (80%)	80 (20%)
Late (D10)	505 (68.3%)	234 (31.7%)
sh*Jmjd3*	Up	Down
Early (D5)	644 (43.7%)	829 (56.3%)
Late (D10)	718 (38.5%)	1145 (61.5%)

(q value < 0.1, fold change > 1.5)

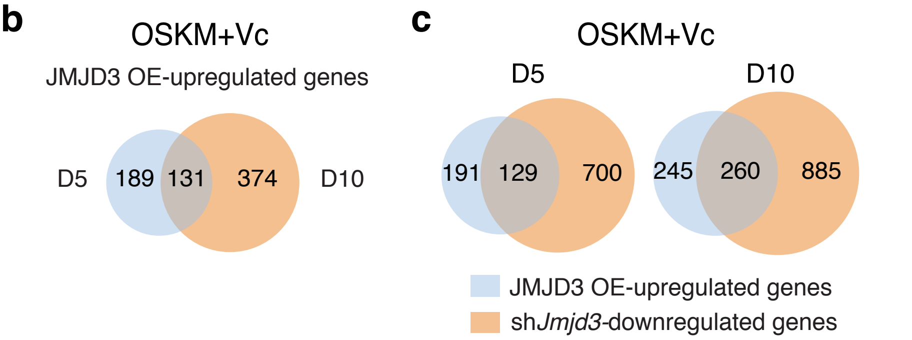

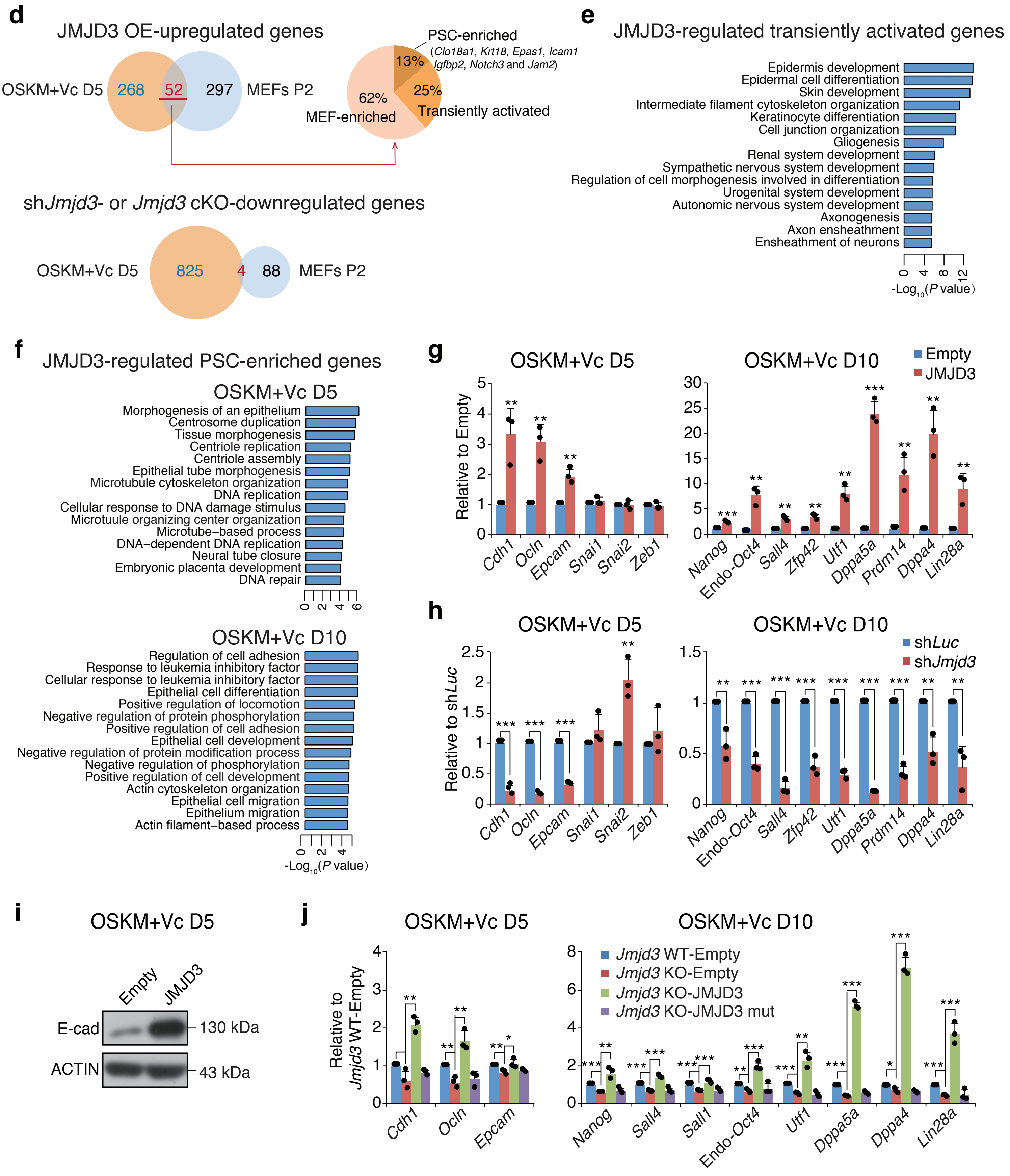

Supplementary Figure 5 JMJD3 promotes epithelial and pluripotency gene expression.

(**a**) Number and percentage of significantly (q value < 0.1, fold change > 1.5) upregulated and downregulated genes at days 5 and 10 upon JMJD3 OE or knockdown.
(**b**,**c**) Venn diagram analysis of genes significantly upregulated upon JMJD3 OE at days 5 and 10 (**b**), and genes significantly upregulated upon JMJD3 OE and downregulated by sh*Jmjd3* at days 5 and 10 (**c**).
(**d**) Venn diagram analysis of genes upregulated upon JMJD3 OE in OSKM-reprograming at day 5 and in P2 MEFs (upper left panel), and genes downregulated by sh*Jmjd3* or *Jmjd3* cKO in OSKM-reprogramming and in P2 MEFs (lower panel). The overlapping genes upregulated upon JMJD3 OE were further analyzed according to the expression pattern (upper right panel).
(**e**,**f**) GO analysis of RNA-seq data identifying the top ranked terms of transiently activated (**e**) and PSC-enriched genes (**f**) upregulated upon JMJD3 OE or downregulated by sh*Jmjd3* in OSKM-reprogrammed P2 MEFs at days 5 and/or 10.
(**g**,**h**) RT-qPCR for the indicated epithelial and mesenchymal genes (day 5, left panel) and pluripotency genes (day 10, right panel) in OSKM-reprogrammed P2 MEFs with Empty or JMJD3 (**g**) or sh*Luc* or sh*Jmjd3* (**h**).
(**i**) Western blotting for E-cadherin in OSKM-reprogrammed P2 MEFs with Empty or JMJD3. Experiments were repeated independently twice with similar results.
(**j**) RT-qPCR for the indicated epithelial (day 5, left panel) and pluripotency (day 10, right panel) genes in the indicated OSKM-reprogrammed WT and *Jmjd3* KO P2 MEFs.
Error bars represent the s.e.m.. Data are presented as mean ± s.e.m. from n=3 (**g**,**h**,**j**) biologically independent experiments. Statistical analyses were performed using a two-tailed unpaired Student's t-test (*$P < 0.05$; **$P < 0.01$; ***$P < 0.001$). P values: 0.0088, 0.0031, 0.0036, 0.0009, 0.0026, 0.0015, 0.0017, 0.0017, $8.55x10^{-5}$, 0.0064, 0.0024, 0.0090 (**g**); $9.13x10^{-5}$, $5.42x10^{-7}$, $1.75x10^{-7}$, 0.0055, 0.0079, 0.0002, $3.52x10^{-5}$, 0.0003, $9.29x10^{-6}$, $4.94x10^{-11}$, $6.07x10^{-5}$, 0.0048, 0.0060 (**h**); 0.0015, 0.0044, 0.0034, 0.0051, 0.0404, $1.36x10^{-7}$, 0.0037, $5.01x10^{-5}$, 0.0002, $3.82x10^{-6}$, 0.0009, 0.0028, 0.0009, 0.0002, 0.0019, $3.6x10^{-6}$, $1.91x10^{-6}$, 0.0189, $3.57x10^{-5}$, $3.56x10^{-5}$, 0.0006 (**j**). Source data are provided as a Source Data file.

Supplementary Figure 6

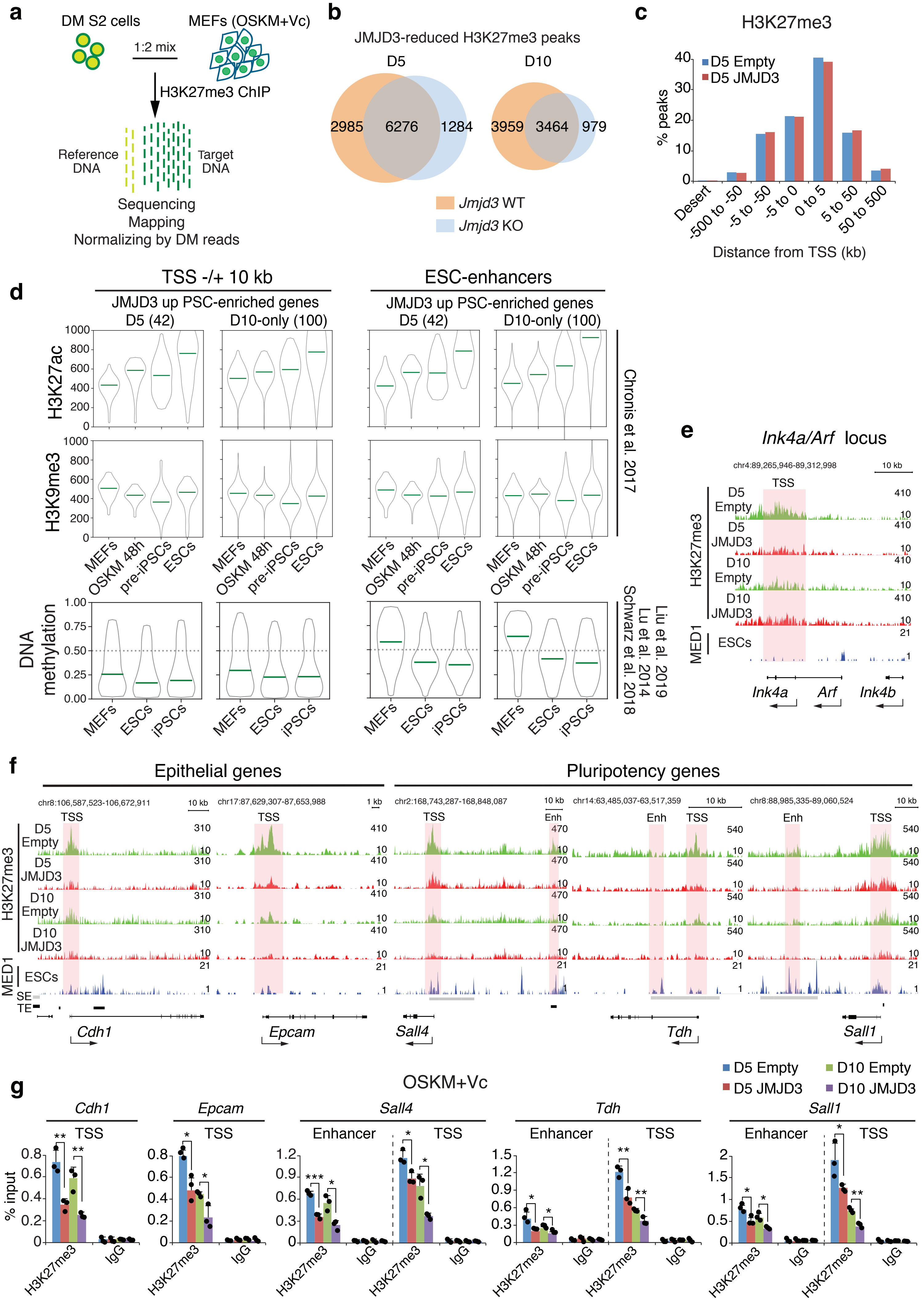

Supplementary Figure 6 Effect of JMJD3 on H3K27me3 demethylation in reprogramming.

(**a**) Schematic representation of the ChIP-seq workflow with reference genome normalization due to the global change of H3K27me3 by JMJD3 OE. S2 cells from Drosophila melanogaster (DM) were used as reference.

(**b**) Venn diagram analysis of H3K27me3 peaks downregulated upon JMJD3 OE (relative to empty vector) in WT and *Jmjd3* KO P2 MEFs transduced with OSKM in medium with Vc at days 5 and 10.

(**c**) Genome distribution of H3K27me3 peaks.

(**d**) Analysis of the dataset (GSE90895) showing violin plots of the levels of H3K27me3 (upper panels) and H3K9me3 (middle panels), and the dataset (GSE106525, GSE112520 and GSE56986) showing violin plots of the level of DNA methylation (lower panels) at TSS and ESC-enhancers of PSC-enriched genes upregulated upon JMJD3 OE from day 5 and day 10, respectively, in the indicated samples.

(**e**-**g**) ChIP-seq data (**e**,**f**) and ChIP-qPCR confirmation (**g**) for H3K27me3 at epithelial (*Cdh1* and *Epcam*) and pluripotency (*Sall4*, *Tdh* and *Sall1*) genes, and the *Ink4a*/*Arf* locus in P2 MEFs transduced with OSKM and Empty or JMJD3 at days 5 and 10. MED1 peaks in ESCs (from GSE22562) were used as reference to indicate potential enhancer sites. SE, super-enhancer; TE, typical enhancer. Shaded regions represent TSS or enhancer.

Error bars represent the s.e.m.. Data are presented as mean ± s.e.m. from n=3 (**g**) biologically independent experiments. Statistical analyses were performed using a two-tailed unpaired Student's t-test (*$P < 0.05$; **$P < 0.01$; ***$P < 0.001$). P values: 0.0051, 0.0054, 0.0133, 0.0284, 0.0003, 0.0128, 0.0175, 0.0115, 0.0267, 0.0203, 0.0095, 0.0095, 0.0209, 0.0105, 0.0482, 0.0017 (**g**). Source data are provided as a Source Data file.

Supplementary Figure 7

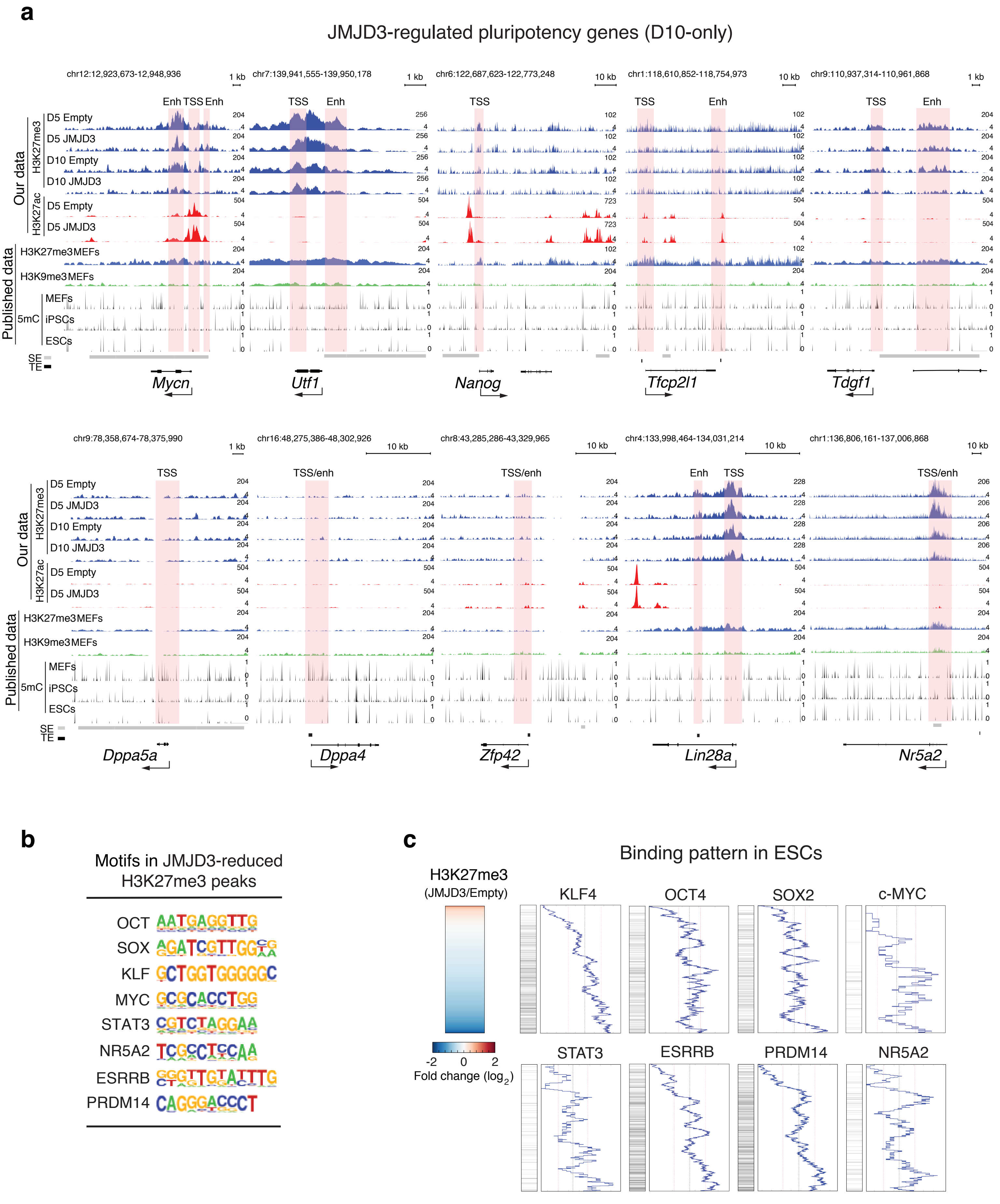

Supplementary Figure 7 H3K27me3 demethylation by JMJD3 at pluripotency loci.

(**a**) Analysis of our H3K27me3 and H3K27ac ChIP-seq dataset in OSKM-reprogrammed P2 MEFs with Empty or JMJD3 at day 5 and/or day 10, and analysis of published datasets for H3K27me3 and H3K9me3 in MEFs (GSE90895) and DNA methylation in MEFs, iPSCs and ESCs (GSE106525, GSE112520 and GSE56986), at the indicated pluripotency gene loci. SE, super-enhancer; TE, typical enhancer. Shaded regions represent TSS or enhancer.

(**b**) De novo DNA sequence motifs of many pluripotency factors detected in H3K27me3 demethylated regions by HOMER.

(**c**) Correlation between H3K27me3 demethylation induced by JMJD3 OE and the binding of the indicated pluripotency factors in ESCs (from GSE11431, GSE19019 and GSE25409). Binding density of the indicated factors was measured using a moving window.

Supplementary Figure 8

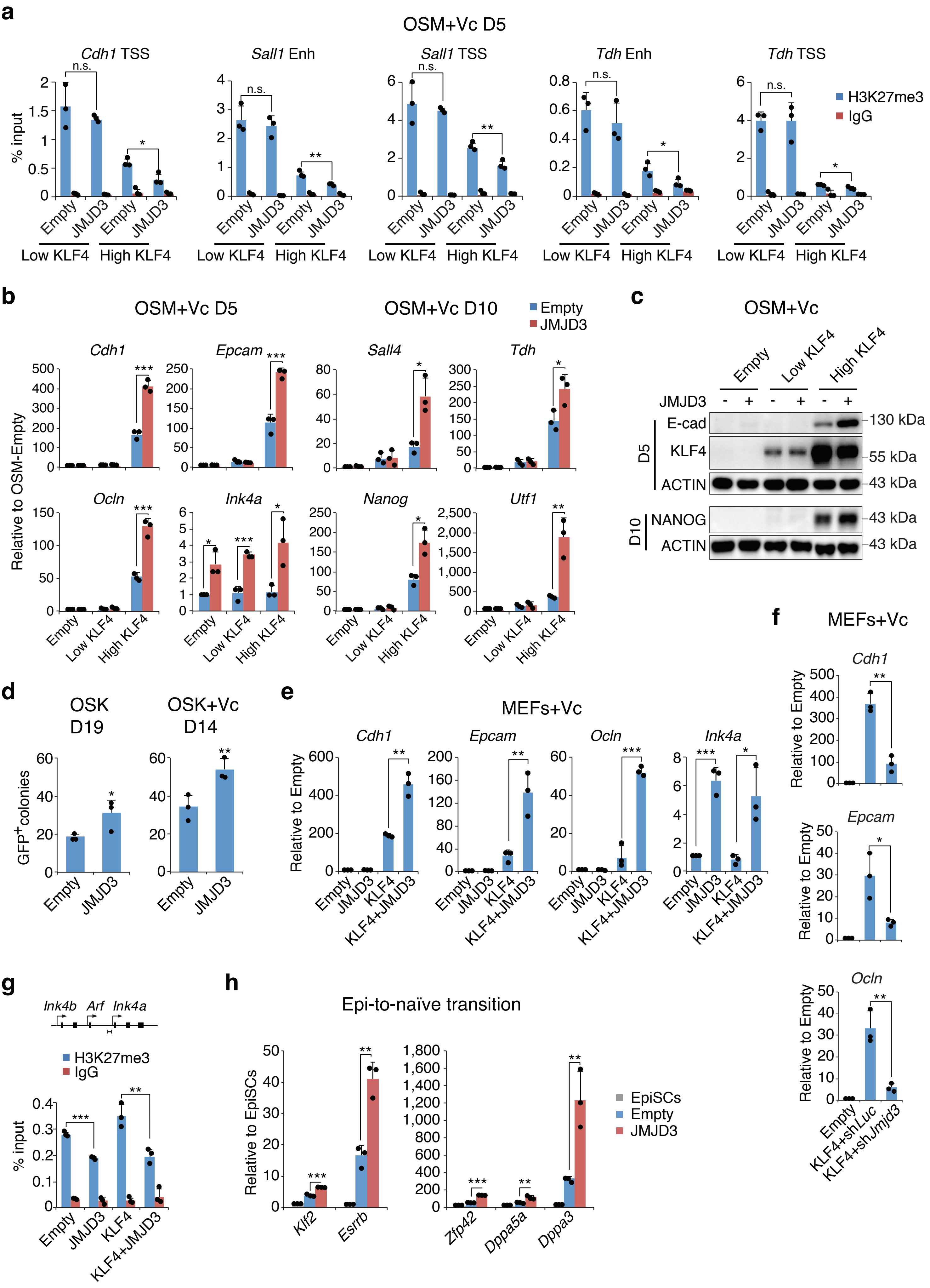

Supplementary Figure 8 JMJD3 and KLF4 cooperatively activate gene expression in multiple cell fate transitions.

(**a**) ChIP-qPCR for H3K27me3 at the indicated loci in P2 MEFs reprogrammed with OSM and low or high level of KLF4 plus Empty or JMJD3.

(**b**) RT-qPCR for epithelial genes (*Cdh1*, *Epcam* and *Ocln*) and *Ink4a* at day 5 (left panels), and pluripotency genes (*Sall4*, *Tdh*, *Nanog* and *Utf1*) at day 10 (right panels) in P2 MEFs reprogrammed with OSM and no KLF4, low KLF4, or high KLF4 plus Empty or JMJD3.

(**c**) Western blotting for E-cadherin, KLF4 (day 5) and NANOG (day 10) in P2 MEFs transduced with OSM and low or high level of KLF4 plus Empty or JMJD3.

(**d**) Number of GFP^+ colonies at day 19 (without Vc) and day 14 (with Vc) in OSK-reprogrammed P2 MEFs with Empty or JMJD3.

(**e**) RT-qPCR for epithelial genes (*Cdh1*, *Epcam* and *Ocln*) and *Ink4a* in P2 MEFs transduced with KLF4 and JMJD3, either alone or in combination, at day 5.

(**f**) RT-qPCR for epithelial genes (*Cdh1*, *Epcam* and *Ocln*) in P2 MEFs transduced with KLF4 and sh*Luc* or sh*Jmjd3* at day 5.

(**g**) ChIP-qPCR for H3K27me3 at the *Ink4a*/*Arf* locus in P2 MEFs transduced with KLF4 and JMJD3, either alone or in combination, at day 5 in medium with Vc.

(**h**) RT-qPCR for naïve ESC-enriched pluripotency genes of the same experiments in Figure 4g.

Error bars represent the s.e.m.. Data are presented as mean ± s.e.m. from n=3 (**a**,**b**,**d**-**h**) biologically independent experiments. Statistical analyses were performed using a two-tailed unpaired Student's *t*-test (*$P < 0.05$; **$P < 0.01$; ***$P < 0.001$). *P* values: 0.0169, 0.0081, 0.0068, 0.0470, 0.0273 (**a**); 0.0003, 0.0009, 0.0006, 0.0144, 0.0008, 0.0237 (D5), 0.0103, 0.0351, 0.0101, 0.0054 (D10) (**b**); 0.0261, 0.0091(**d**); 0.0015, 0.0083, 0.0003, 0.0006, 0.0197 (**e**); 0.0012, 0.0237, 0.0044 (**f**); 0.0002, 0.0077 (**g**); 0.0002, 0.0026, 1.45×10^{-6}, 0.0026, 0.0098 (**h**). Experiments were repeated independently twice (**c**) with similar results. Source data are provided as a Source Data file.

Supplementary Figure 9

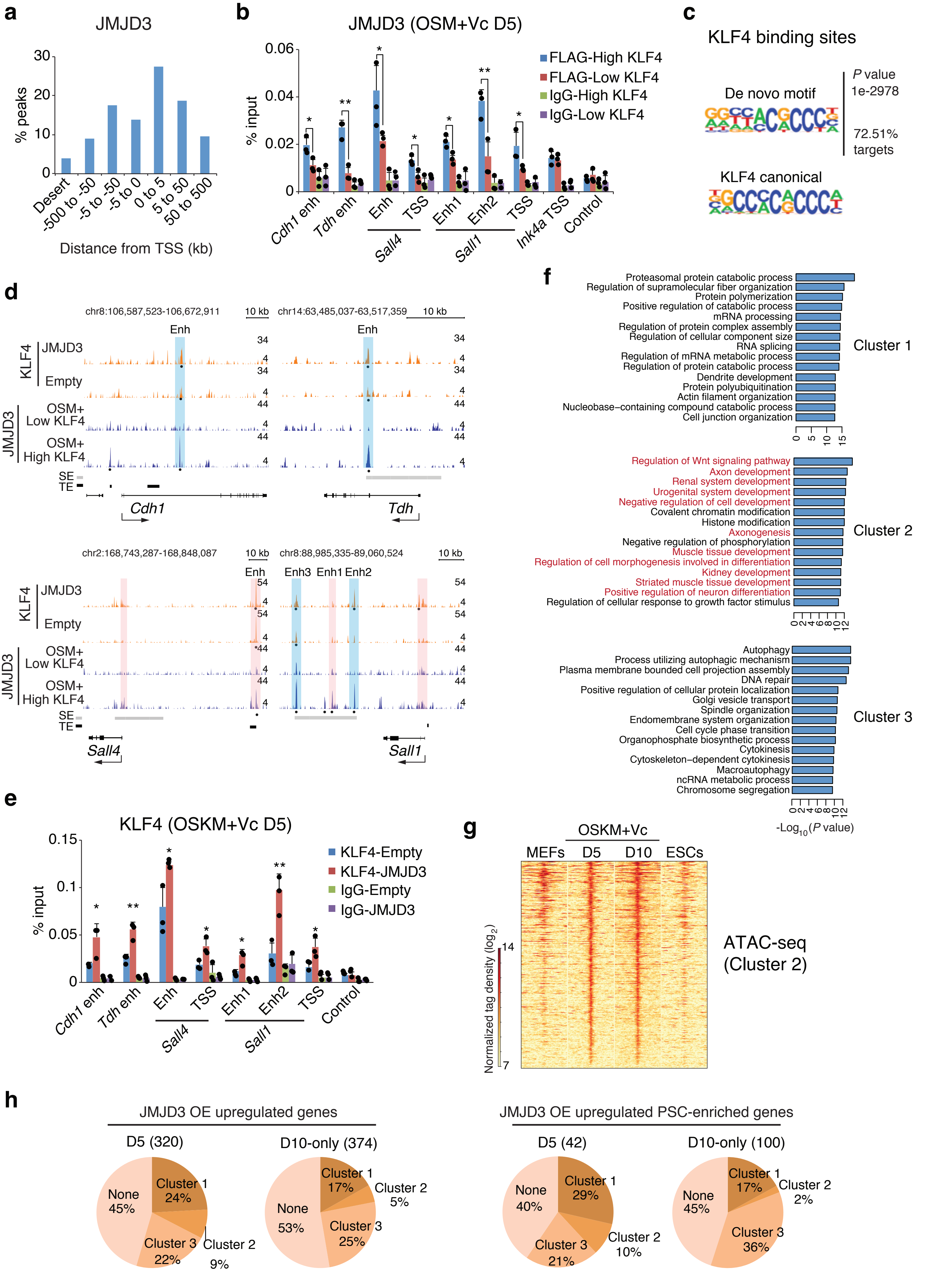

Supplementary Figure 9 Interrelationship between genome-wide JMJD3 and KLF4 binding.

(**a**) Genome distribution of JMJD3 ChIP-seq peaks in P2 MEFs transduced with OSKM and JMJD3 at day 5 in medium with Vc.
(**b**) ChIP-qPCR for FLAG-tagged JMJD3 at the indicated loci in P2 MEFs transduced with OSM and low or high level of KLF4 plus JMJD3 in medium with Vc at day 5. *Ink4a* TSS was used as a KLF4-independent control. Control is a locus in gene desert regions where JMJD3 does not bind.
(**c**) De novo motifs found in KLF4 binding sites by HOMER.
(**d**) KLF4 tag density from P2 MEFs transduced with OSKM and Empty or JMJD3 at day 5, and JMJD3 tag density from MEFs transduced with OSM and low or high level of KLF4 at day 5 at *Cdh1*, *Tdh*, *Sall4* and *Sall1*.
(**e**) ChIP-qPCR for KLF4 binding at the indicated loci in P2 MEFs transduced with OSKM and Empty or JMJD3 in medium with Vc at day 5. Control is a locus in gene desert regions where KLF4 does not bind.
(**f**) GO analysis identifying the top ranked terms of genes around the peaks in each cluster (1, 2 and 3 in Figure 5e).
(**g**) Tag density heatmaps for ATAC-seq of MEFs, OSKM-reprogramming plus Vc at days 5 and 10, and ESCs (OG2 ESCs) in the peaks of cluster 2 in Figure 5e.
(**h**) Overlapping of all genes (left panels) or only PSC-enriched genes (right panels) upregulated upon JMJD3 OE with the genes bound by KLF4 and/or JMJD3 (cluster 1, 2 and 3 in Figure 5e).
Error bars represent the s.e.m.. Data are presented as mean ± s.e.m. from n=3 (**b**,**e**) biologically independent experiments. Statistical analyses were performed using a two-tailed unpaired Student's t-test (*$P < 0.05$; **$P < 0.01$; ***$P < 0.001$). P values: 0.0166, 0.0011, 0.0296, 0.0104, 0.0102, 0.0066, 0.0332 (**b**); 0.0487, 0.0065, 0.0429, 0.0301, 0.0210, 0.0094, 0.0424 (**e**). Source data are provided as a Source Data file.

Supplementary Figure 10

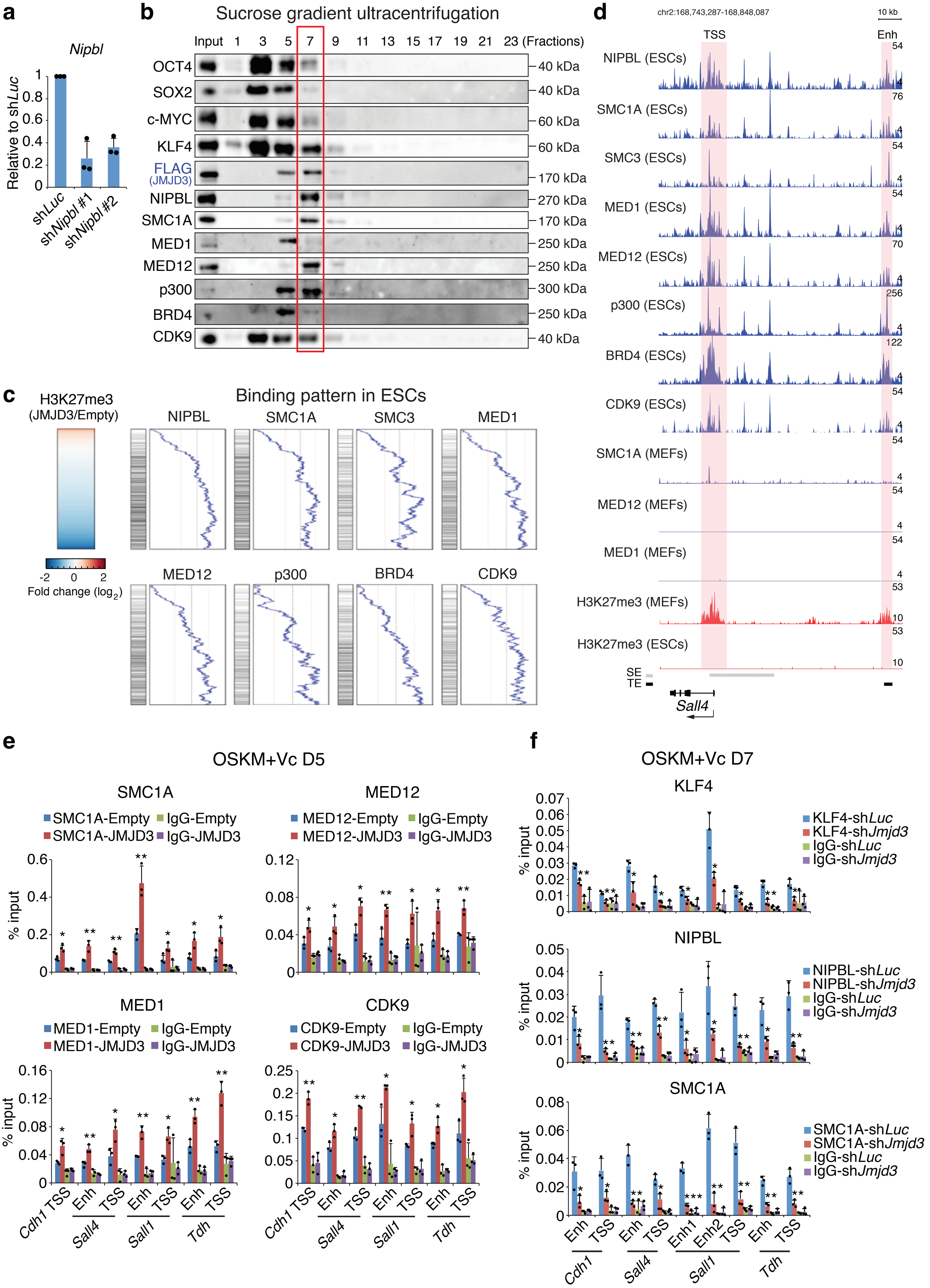

Supplementary Figure 10 JMJD3 promotes Cohesin-Mediator loading on target sites.

(**a**) RT-qPCR analysis showing the knockdown efficiency of sh*Nipbl* in P2 MEFs transduced with OSKM.

(**b**) Nuclear lysates from P2 MEFs transduced with OSKM and FLAG-tagged JMJD3 at day 5 in medium with Vc were sedimented on a 10–30% sucrose gradient by ultracentrifugation and divided into 24 fractions. The gradients with odd numbers were analyzed by western blotting with the indicated antibodies.

(**c**) Correlation between H3K27me3 demethylation by JMJD3 and the binding of the indicated factors in ESCs (from GSE22562, GSE56098, GSE67944 and GSE44286). The binding density of the indicated factors was measured using a moving window.

(**d**) ChIP-seq data for the indicated factor binding on *Sall4* locus in ESCs and MEFs (from GSE22562, GSE56098, GSE44286, GSE67944 and GSE12241).

(**e**) ChIP-qPCR for the binding of SMC1A, MED12, MED1 and CDK9 at the indicated loci in P2 MEFs transduced with OSKM and Empty or JMJD3 in medium with Vc at day 5.

(**f**) ChIP-qPCR for the binding of KLF4, NIPBL and SMC1A at the indicated loci in P2 MEFs transduced with OSKM and sh*Luc* or sh*Jmjd3* in medium with Vc at day 7.

Error bars represent the s.e.m.. Data are presented as mean ± s.e.m. from n=3 (**a**,**e**,**f**) biologically independent experiments. Statistical analyses were performed using a two-tailed unpaired Student's *t*-test ($^*P < 0.05$; $^{**}P < 0.01$; $^{***}P < 0.001$). P values: 0.0117, 0.0099, 0.0031, 0.0080, 0.0381, 0.0273, 0.0335 (SMC1A), 0.0329, 0.0467, 0.0161, 0.0091, 0.0160, 0.0145, 0.0032 (MED12), 0.0175, 0.0068, 0.0250, 0.0020, 0.0115, 0.0064, 0.0020 (MED1), 0.0013, 0.0273, 0.0014, 0.0206, 0.0276, 0.0211, 0.0192 (CDK9) (**e**); 0.0016, 0.0085, 0.0199, 0.0246, 0.0384, 0.0103, 0.0280, 0.0027, 0.0115 (KLF4), 0.0235, 0.0082, 0.0029, 0.0023, 0.0425, 0.0322, 0.0032, 0.0177, 0.0053 (NIPBL), 0.0323, 0.0225, 0.0015, 0.0120, 0.0007, 0.0015, 0.0037, 0.0018, 0.0057 (SMC1A) (**f**). Experiments were repeated independently twice (**b**) with similar results. Source data are provided as a Source Data file.

Supplementary Figure 11

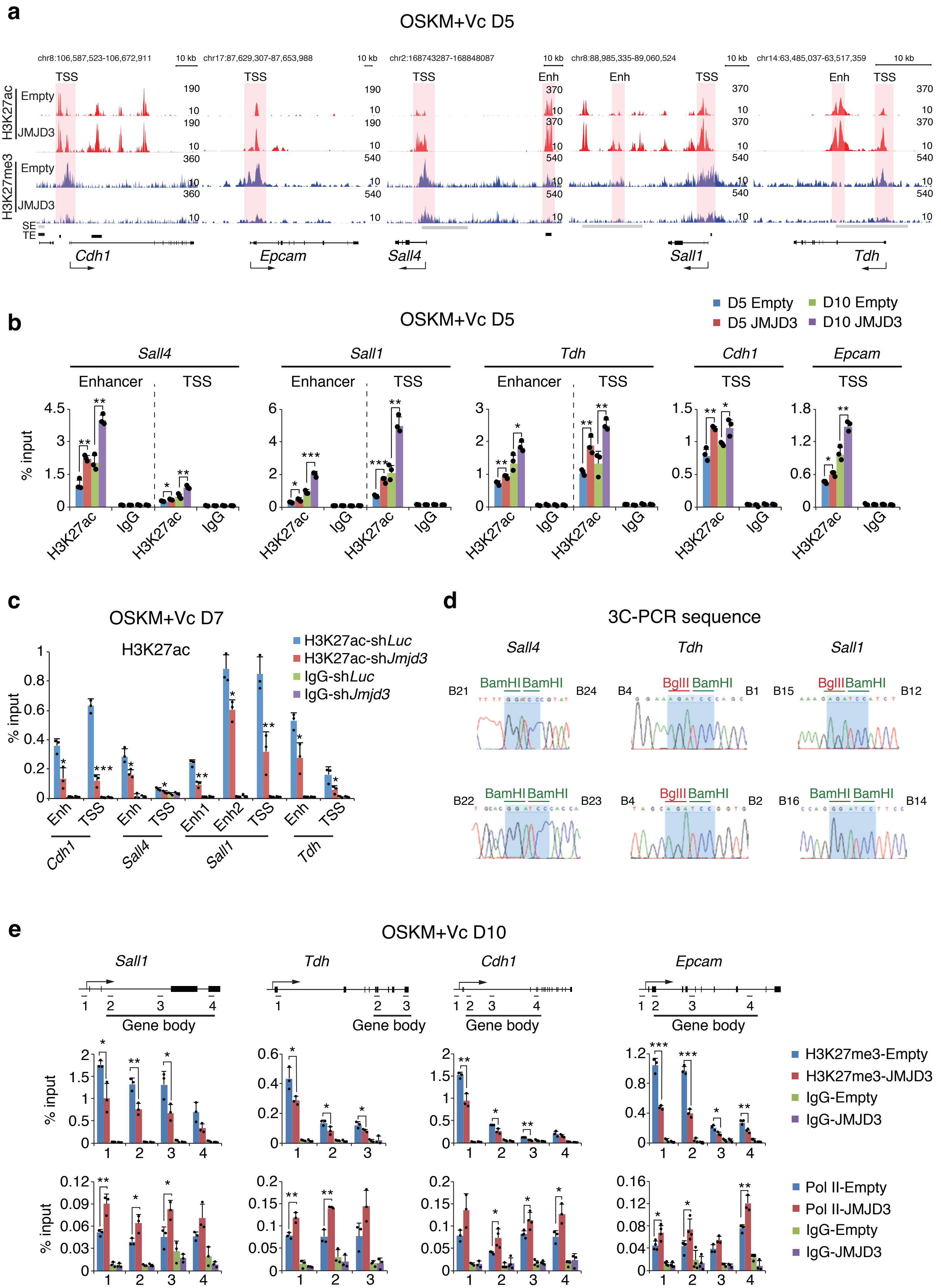

Supplementary Figure 11 JMJD3 facilitates H3K27ac deposition and transcriptional elongation.

(**a**,**b**) H3K27ac and H3K27me3 tag density from ChIP-seq data at *Cdh1*, *Epcam*, *Sall4*, *Sall1* and *Tdh* loci (**a**), and ChIP-qPCR validation for H3K27ac change at the indicated loci (**b**) in P2 MEFs transduced with OSKM and Empty or JMJD3.

(**c**) ChIP-qPCR for H3K27ac at the indicated loci in OSKM-reprogrammed P2 MEFs with sh*Luc* or sh*Jmjd3*.

(**d**) Sequencing results for validation of the ligation of interacted genomic fragments.

(**e**) ChIP-qPCR for H3K27me3 and Pol II binding at the TSS and gene body of the indicated genes in OSKM-reprogrammed P2 MEFs with Empty or JMJD3.

Error bars represent the s.e.m.. Data are presented as mean ± s.e.m. from n=3 (**b**,**c**,**e**) biologically independent experiments. Statistical analyses were performed using a two-tailed unpaired Student's t-test (*P < 0.05; **P < 0.01; ***P < 0.001). P values: 0.0015, 0.0014, 0.0111, 0.0054, 0.0307, 0.0005, 0.0007, 0.0020, 0.0097, 0.0486, 0.0071, 0.0076, 0.0018, 0.0337, 0.0245, 0.0047 (**b**); 0.0124, 0.0001, 0.0208, 0.0420, 0.0011, 0.0168, 0.0074, 0.0180, 0.0430 (**c**); 0.0201, 0.0069, 0.0370, 0.0574 (*Sall1*-H3K27me3), 0.0087, 0.0268, 0.0239, 0.0919 (*Sall1*-Pol II), 0.0353, 0.0458, 0.0433 (*Tdh*-H3K27me3), 0.0065, 0.0022,0.0676 (*Tdh*-Pol II),0.0038, 0.0151, 0.0013 (*Cdh1*-H3K27me3), 0.0188, 0.0167, 0.0302 (*Cdh1*-Pol II), 0.0007, 0.0003, 0.0225, 0.0070 (*Epcam*-H3K27me3), 0.0290, 0.0304, 0.0517, 0.0080 (*Epcam*-Pol II) (**e**). Source data are provided as a Source Data file.

Supplementary Figure 12

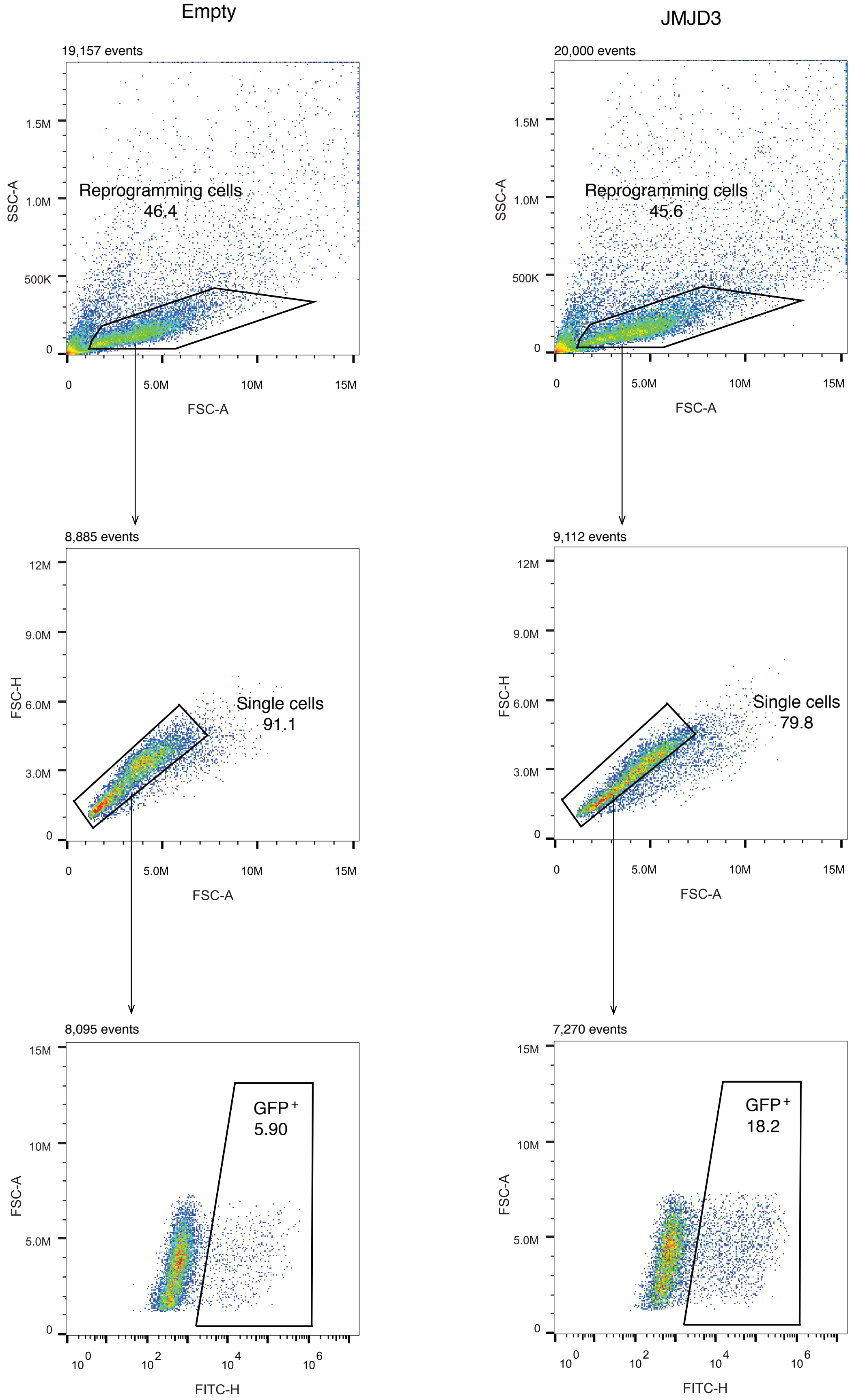

Supplementary Figure 12 Sequential gating strategy for flow cytometry analysis in Figure 4g.
Flow cytometry analysis for *Oct4*-GFP-positive (FITC) cells during Epi-to-naïve PSC transition with Empty or JMJD3.

Supplementary Figure 13

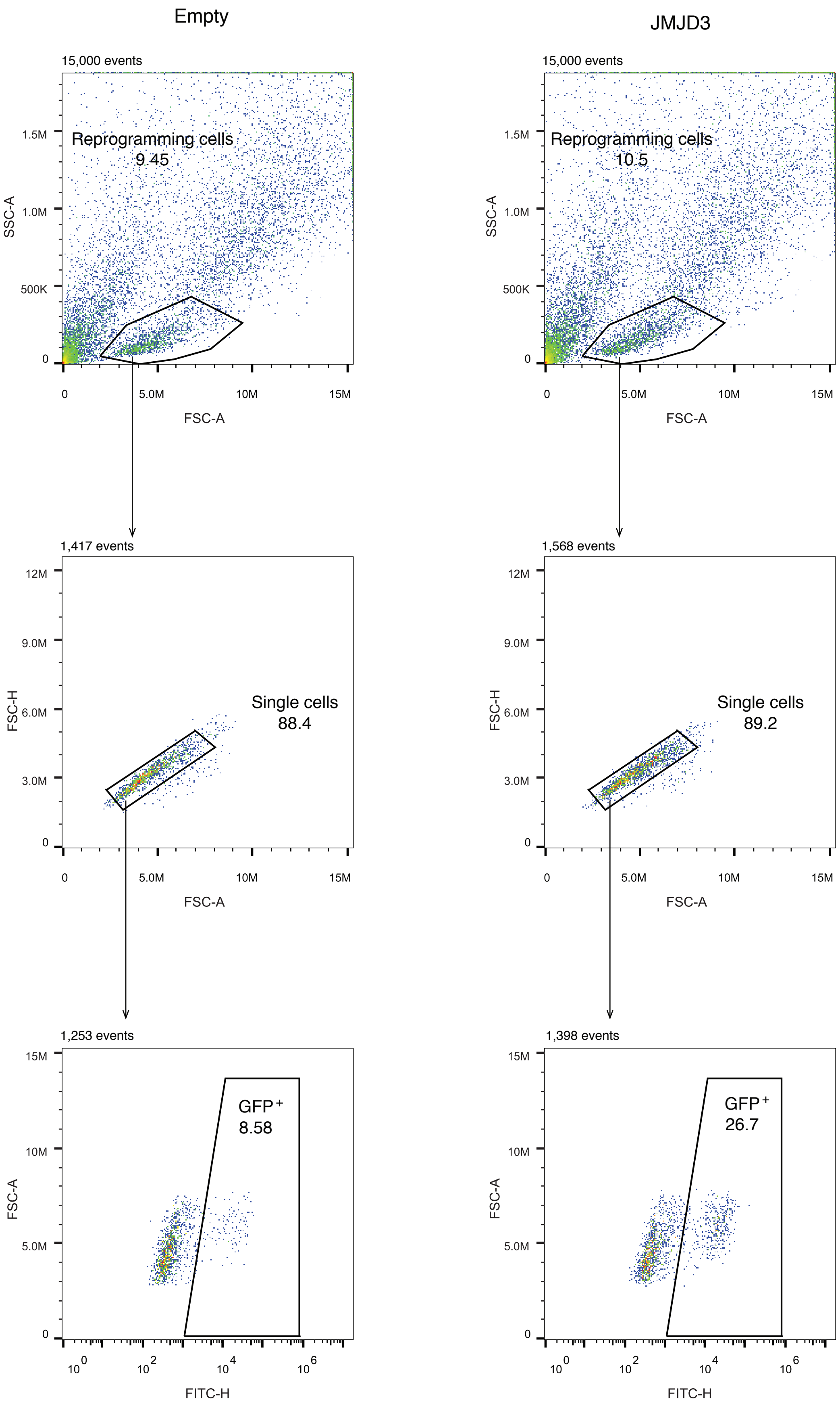

Supplementary Figure 13 Sequential gating strategy for flow cytometry analysis in Figure 4h.
Flow cytometry analysis for Rex1GFPd2-positive (FITC) cells during Epi-to-naïve PSC transition with Empty or JMJD3.

Supplementary Figure 14

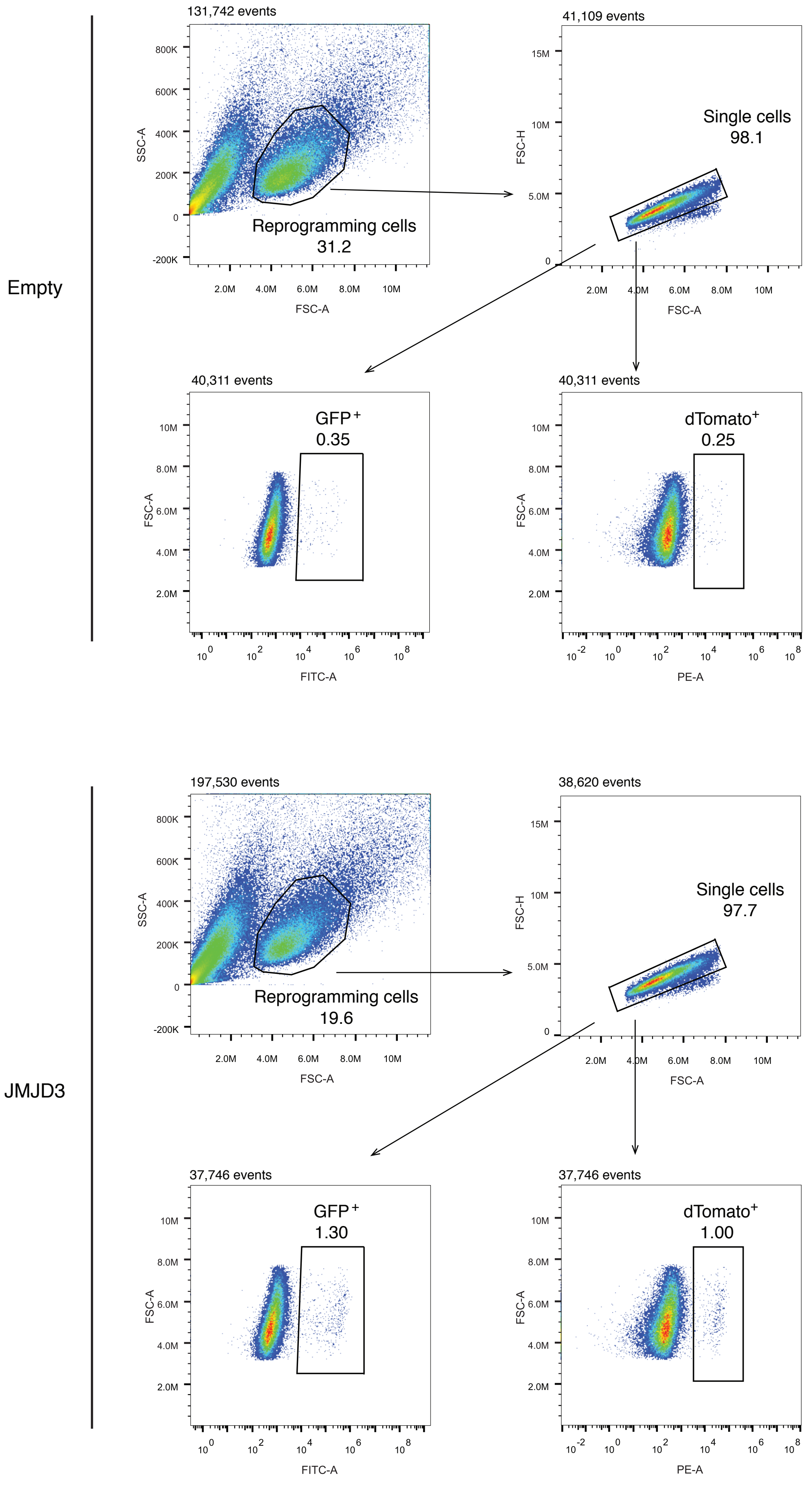

Supplementary Figure 14 The sequential gating strategy for flow cytometry analysis in Supplementary Figure 1d.

Flow cytometry analysis for *Oct4*-GFP-positive (FITC) or *Dppa5a*-dTomato-positive (PE) cells during OSKM-driven reprogramming with Empty or JMJD3 at day 11.

Supplementary Figure 15

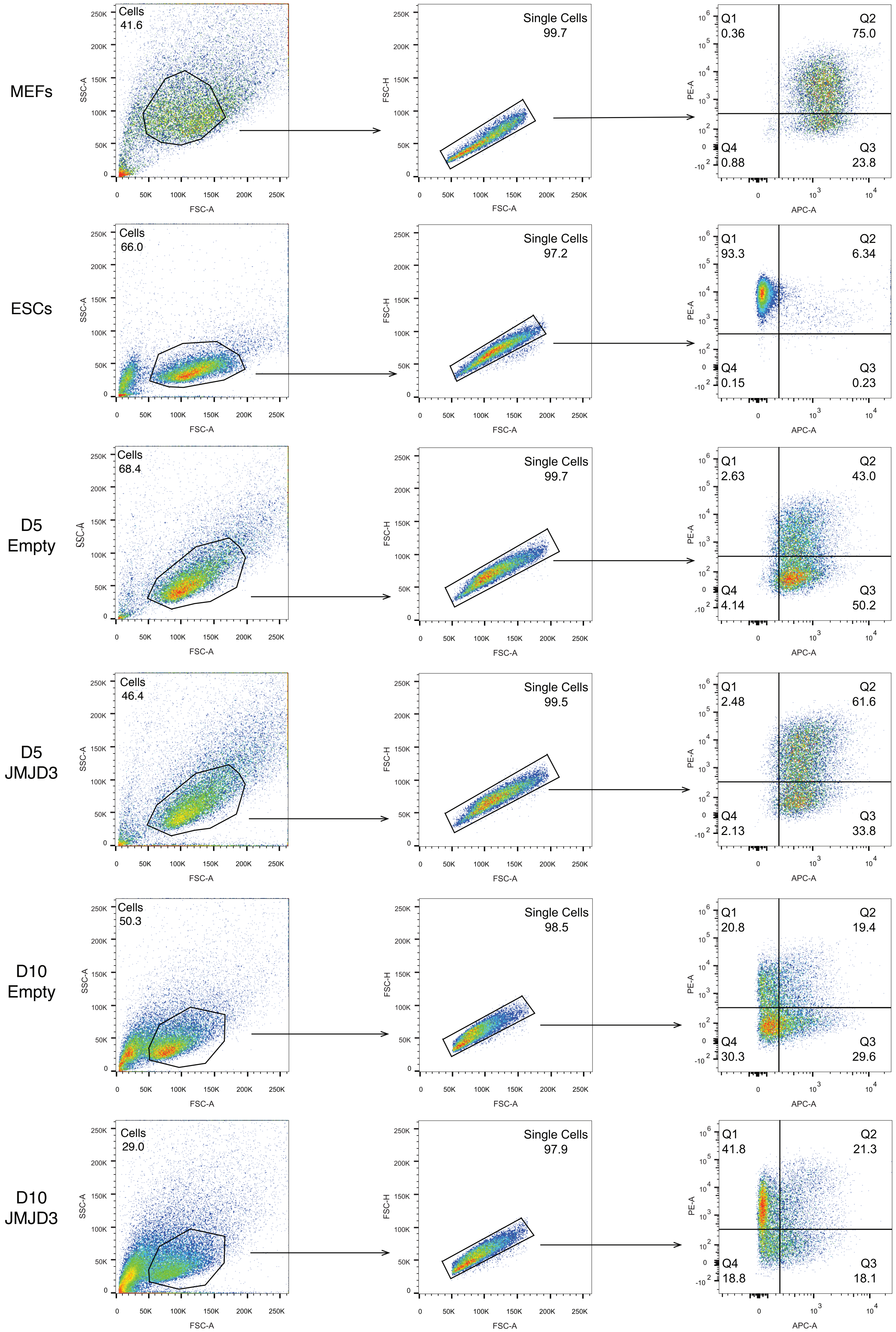

Supplementary Figure 15 The sequential gating strategy for flow cytometry analysis in Supplementary Figure 1k. Flow cytometry analysis for ICAM1 (PE) and CD44 (APC) staining in MEFs, ESCs and OSKM-reprogrammed MEFs with Empty or JMJD3 at days 5 and 10.
