# Supplementary material for: JMJD3 acts in tandem with KLF4 to facilitate reprogramming to pluripotency
Source: Nat Commun. 2020 Oct 8;11:5061. doi: 10.1038/s41467-020-18900-z (PMC7545202; doi:10.1038/s41467-020-18900-z)
Supplement: Supplementary file 3 — Reporting Summary [file 41467_2020_18900_MOESM3_ESM.pdf]

nature research

Corresponding author(s): Baoming Qin and Miguel A. Esteban

Last updated by author(s): Aug 13, 2020

Reporting Summary

Nature Research wishes to improve the reproducibility of the work that we publish. This form provides structure for consistency and transparency in reporting. For further information on Nature Research policies, see Authors & Referees and the Editorial Policy Checklist .

Statistics

For all statistical analyses, confirm that the following items are present in the figure legend, table legend, main text, or Methods section.

n/a	Confirmed	
☐	☒	The exact sample size (*n*) for each experimental group/condition, given as a discrete number and unit of measurement
☐	☒	A statement on whether measurements were taken from distinct samples or whether the same sample was measured repeatedly
☐	☒	The statistical test(s) used AND whether they are one- or two-sided<br>*Only common tests should be described solely by name; describe more complex techniques in the Methods section.*
☐	☒	A description of all covariates tested
☒	☐	A description of any assumptions or corrections, such as tests of normality and adjustment for multiple comparisons
☐	☒	A full description of the statistical parameters including central tendency (e.g. means) or other basic estimates (e.g. regression coefficient) AND variation (e.g. standard deviation) or associated estimates of uncertainty (e.g. confidence intervals)
☐	☒	For null hypothesis testing, the test statistic (e.g. *F*, *t*, *r*) with confidence intervals, effect sizes, degrees of freedom and *P* value noted<br>*Give P values as exact values whenever suitable.*
☒	☐	For Bayesian analysis, information on the choice of priors and Markov chain Monte Carlo settings
☒	☐	For hierarchical and complex designs, identification of the appropriate level for tests and full reporting of outcomes
☐	☒	Estimates of effect sizes (e.g. Cohen's *d*, Pearson's *r*), indicating how they were calculated

Our web collection on statistics for biologists contains articles on many of the points above.

Software and code

Policy information about availability of computer code

Data collection	In general, results presented as mean ± s.e.m. were collected and calculated using Microsoft Excel 2010. No samples were excluded for any analysis.<br>For AP staining, colony number counting data were collected with Image Pro Plus 6.0<br>For RT-qPCR, data were collected with Real Time PCR Software v2.4<br>For western blotting, images were collected with FusionCapt Advance Solo4.16.15<br>For immunofluorescence, images were collected with ZEN software v2.0.<br>For flow cytometry, data were collected with BD Accuri C6 Plus software (v1.0.23.1) or BD FACSDiva software (v8.0.1).
Data analysis	Results (RT-qPCR, ChIP-qPCR, colony numbers, statistics for flow cytometry and the quantification of western blotting gels) presented as mean ± s.e.m. were analyzed by Microsoft Excel 2010 and GraphPad Prism 8;.<br>For western blotting, image processing and relative density calculation were analyzed with FusionCapt Advance Solo4.16.15.<br>For flow cytometry, data were analyzed by Flowjo (v10.4).<br>For RNA-seq, data were analysed with Ensembl v76, bowtie2 v2.4.1, RSEM v1.2.18, EDASeq v2.0.0, Deseq2 v1.8.1 and Goseq v1.20.0<br>For ChIP-seq, data were analysed with DFilter v1.6, MACS2 v2.2.5, HOMER v4.10.3 and glbase (Hutchins AP. et al. Cell Regen. 2014;3(1)).<br>For ATAC-seq, data were analysed with bowtie2 v2.4.1, samtools v1.9 and grep v2.20.

For manuscripts utilizing custom algorithms or software that are central to the research but not yet described in published literature, software must be made available to editors/reviewers. We strongly encourage code deposition in a community repository (e.g. GitHub). See the Nature Research guidelines for submitting code & software for further information.

Data

Policy information about availability of data

All manuscripts must include a data availability statement. This statement should provide the following information, where applicable:

- Accession codes, unique identifiers, or web links for publicly available datasets
- A list of figures that have associated raw data
- A description of any restrictions on data availability

RNA-seq, ATAC-seq and ChIP-seq data are available in GEO database under the accession number GSE75005. Published data utilized in this study are under the accession number: GSE93027 (RNA-seq for PSCs including ESCs and iPSCs), GSE22562 (MED1, MED12, NIPBL, SMC1A and SMC3 in ESCs), GSE56098 (p300 in ESCs), GSE19019 (NR5A2 in ESCs), GSE25409 (PRDM14 in ESCs), GSE11431 (ESRRB and STAT3 in ESCs), GSE90895 (SOX2, KLF4, OCT4 and c-MYC in ESCs; H3K27me3, H3K27ac and H3K9me3 in MEFs, OSKM reprogramming at 48 hours, pre-iPSCs and ESCs), GSE44286 (CDK9 in ESCs), GSE67944 (BRD4 in ESCs), GSE106525 (WGBS in MEFs and iPSCs), GSE112520 (WGBS in ESCs) and GSE56986 (WGBS in ESCs). Source data are provided with this paper. Source data underlying Figs. 1a-g, 2a, c-h, 4a-h, 6b-f, i, j and Supplementary Figs. 1a-f, i-k, 2a-c, 3a, 4a-m, 5g-j, 6g, 8a-h, 9b, e, 10a, b, e, f, 11b, c, e are provided as a Source Data file. The gating strategies for all flow cytometry experiments are provided in Supplementary Fig. 12-15. A Reporting Summary for this article is available as a Supplementary Information file. All other data supporting the findings of this study are available from the corresponding authors upon reasonable request.

Field-specific reporting

Please select the one below that is the best fit for your research. If you are not sure, read the appropriate sections before making your selection.

[x] Life sciences   [ ] Behavioural & social sciences   [ ] Ecological, evolutionary & environmental sciences

For a reference copy of the document with all sections, see nature.com/documents/nr-reporting-summary-flat.pdf

Life sciences study design

All studies must disclose on these points even when the disclosure is negative.

Sample size	No statistical method was used to predetermine sample size. Experiments were performed at least three times to obtain statistical significance. Cell numbers of relative experiments in this study were determined based on our experience and previous studies in this field.
Data exclusions	No data were excluded in this study.
Replication	Fig. 1a-g, 2d-g, 4b, 4d, 4g, 4h, 6e, 6f and Supplementary Fig. 1a, 1c, 1d, 1i-k, 2c, 4a-e, 4g, 4i, j (right), k-m, 5g, 5h, 5j, 8b, 8d-f, 8h, 10a were repeated three times independently (biological replicates with three technical replicates each); Fig. 4c, 4f, 6d, 6j and Supplementary Fig. 6g, 8a, 8g, 9b, 9e, 10e, 10f, 11b, 11c, 11e were repeated three times independently (biological replicates with two technical replicates each); Fig. 2a (lower), 2c (right), 2h (right) and Supplementary Fig. 2a (right), 3a (right) were repeated three times (biological replicates with one technical replicate); Supplementary Fig. 1e, 1f were performed once (with three technical replicates).<br><br>The RNA-seq in Fig. 3a was performed once except for the ESCs (three times); the ChIP-seq in Fig. 3d, 5a, 5c, 6h were performed once; Supplementary Fig. 1g was performed once; the ATAC-seq in Supplementary Fig. 9g was performed twice.<br><br>For reproducibility of representative figures: Fig. 2a (upper), 2c (left), 2h (left), 4a, 6b and Supplementary Fig. 1b, 2a (left), 3a (left), 3b are representative of at least three independent experiments; Fig. 4e, 6c, 6i and Supplementary Fig. 4f, 5i, 8c, 10b are representative of two independent experiments; Supplementary Fig. 2b, 4h, 4j (left) were performed once to confirm protein/gene expression.
Randomization	For chimera experiments, mice were selected randomly for blastocysts injection. For MEF isolation, the embryos were selected based on the genotype we needed. Randomization was not relevant to other experiments, but control and experimental samples were always processed in parallel to control covariates.
Blinding	The investigators were blinded to RNA-seq, ChIP-seq and ATAC-seq analysis, and all other data collection and/or analysis.

Reporting for specific materials, systems and methods

We require information from authors about some types of materials, experimental systems and methods used in many studies. Here, indicate whether each material, system or method listed is relevant to your study. If you are not sure if a list item applies to your research, read the appropriate section before selecting a response.

Materials & experimental systems

n/a	Involved in the study	
	x	Antibodies
	x	Eukaryotic cell lines
x		Palaeontology
	x	Animals and other organisms
x		Human research participants
x		Clinical data

Methods

n/a	Involved in the study	
	x	ChIP-seq
	x	Flow cytometry
x		MRI-based neuroimaging

Antibodies

Antibodies used

The following antibodies were used as primary antibodies for western blotting, immunofluorescence, co-immunoprecipitation, immunoprecipitation-MS and flow cytometry.
anti-FLAG (Sigma F1804) western blotting 1:1000, immunofluorescence 1:200
anti-FLAG M2 magnetic beads (Sigma, M8823) co-immunoprecipitation and immunoprecipitation-MS 30 µl
anti-SOX2 (R&D MAB2018) western blotting 1:1000
anti-KLF4 (R&D AF3158) western blotting 1:1000
anti-OCT3/4 (Santa Cruz sc-8628) western blotting 1:1000
anti-c-MYC (R&D AF3696) western blotting 1:1000
anti-NANOG(BETHYL A300-397A) western blotting 1:1000, immunofluorescence 1:200
anti-P16 (Santa Cruz sc-1207) western blotting 1:1000
anti-NIPBL (BETHYL A301-779A) western blotting 1:1000, co-immunoprecipitation 10 µg
anti-MED1 (BETHYL A300-793) western blotting 1:1000
anti-MED12 (BETHYL A300-774) western blotting 1:1000
anti-SMC1A (BETHYL A300-055A) western blotting 1:1000
anti-CDK9 (Santa Cruz sc-484) western blotting 1:1000
anti-p300 (Santa Cruz sc-585) western blotting 1:1000
anti-BRD4 (BETHYL A301-985A100) western blotting 1:1000
anti-RbBP5 (BETHYL A300-109A) western blotting 1:1000
anti-RING1B (Cell Signaling Technology 5694S) western blotting 1:1000
anti-H3K27me3 (Millipore 07-449) western blotting 1:1000, immunofluorescence 1:200
anti-Histone H3 (Abcam ab1791) western blotting 1:3000
anti-PHF20 (Cell Signaling Technology 3934) western blotting 1:1000
anti-ACTIN (Sigma A2066) western blotting 1:1000
and anti-E-cadherin (BD Biosciences 610181) western blotting 1:1000

The following antibodies were used for ChIP:
anti-H3K27me3 (Millipore 07-449, 5 µg/ChIP)
anti-H3K27ac (Abcam ab4729, 5 µg/ChIP)
anti-Pol II (Santa Cruz sc-899, 10 µg/ChIP)
anti-NIPBL (BETHYL A301-779A, 10 µg/ChIP)
anti-MED1 (BETHYL A300-793, 10 µg/ChIP)
anti-MED12 (BETHYL A300-774, 10 µg/ChIP)
anti-SMC1A (BETHYL A300-055, 10 µg/ChIP)
anti-CDK9 (Santa Cruz sc-484, 10 µg/ChIP)
anti-KLF4 (R&D AF3158, 10 µg/ChIP)
anti-FLAG (Sigma F1804, 10 µg/ChIP)
mouse anti-IgG (Beyotime A7028, 5 or 10 µg/ChIP)
rabbit anti-IgG (Beyotime A7016, 5 or 10 µg/ChIP)
goat anti-IgG (Beyotime A7007, 5 or 10 µg/ChIP)

The following antibodies were used for flow cytometry:
anti-CD44-APC (eBiosciences 17-0441, 1:200)
anti-ICAM1-PE (eBiosciences 12-0542, 1:200)

Validation

Anti-FLAG (Sigma F1804; western blotting 1:1000; immunofluorescence 1:200). Validated by 4901 citations (for example Jahn A. et al. EMBO Rep. 2017;18(6):929-946 with western blot and immunofluorescence for human cells).

Anti-FLAG M2 magnetic beads (Sigma M8823; co-immunoprecipitation and immunoprecipitation-MS 30 µl). Validated by 180 citations (for example Mor N. et al. Cell Stem Cell. 2018;23(3):412-425.e10. with co-immunoprecipitation for mouse cells).

Anti-SOX2 (R&D MAB2018; western blotting 1:1000). Validated by the manufacturer with immunocytochemistry and flow cytometry for human, western blot for human, mouse and rat, and also validated by 219 citations (for example Gonnot, F. et al. Cell Cycle. 2019;18(20):2697-2712 with western blot for mouse cells).

Anti-KLF4 (R&D AF3158; western blotting 1:1000). Validated by the manufacturer with ChIP and immunocytochemistry for mouse, and also validated by 82 citations (for example Chuang H.C. et al. Sci. Adv. 2018;4(9) with ChIP for mouse cells).

Anti-OCT3/4 (Santa Cruz sc-8628; western blotting 1:1000). Validated by 76 citations (for example Tahmasebi, S. et al. Proc. Natl. Acad. Sci. USA. 2016;113(44):12360-12367 with western blot for mouse cells).

Anti-c-MYC (R&D AF3696; western blotting 1:1000). Validated by the manufacturer with western blot and immunocytochemistry for mouse and human, ChIP for human, and also validated by 12 citations (for example Morrison G. et al. EMBO J. 2016;35 (3):356-368. with ChIP for mouse cells).

Anti-NANOG (BETHYL A300-397A; western blotting 1:1000; immunofluorescence 1:200). Validated by 81 citations (for example Song, K.H. et al. Nat. Commun. 2020;11(1):562 with western blot and ChIP for human cells).

Anti-P16 (Santa Cruz sc-1207; western blotting 1:1000). Validated by 263 citations (for example Cassidy L.D. et al. Nat. Commun. 2020;11(1):307 with western blot for mouse cells).

Anti-NIPBL (BETHYL A301-779A; western blotting 1:1000). Validated by the manufacturer with western blot for human, and also validated by 21 citations (for example Kagey M.H. et al. Nature 2010;467(7314):430-435 with western blot, immunoprecipitation and ChIP-seq for mouse cells).

Anti-MED1 (BETHYL A300-793; western blotting 1:1000). Validated by the manufacturer with western blot for human and mouse, and also validated by 72 citations (for example Kagey M.H. et al. Nature 2010;467(7314):430-435 with western blot, immunoprecipitation and ChIP-seq for mouse cells).

Anti-MED12 (BETHYL A300-774; western blotting 1:1000). Validated by 42 citations (for example Kagey M.H. et al. Nature 2010;467(7314):430-435 with western blot, immunoprecipitation and ChIP-seq for mouse cells).

Anti-SMC1A (BETHYL A300-055A; western blotting 1:1000). Validated by the manufacturer with western blot and immunohistochemistry on human and mouse, and also validated by 170 citations (for example Kagey M.H. et al. Nature 2010;467(7314):430-435 with western blot, immunoprecipitation and ChIP-seq for mouse cells).

Anti-CDK9 (Santa Cruz sc-484; western blotting 1:1000). Validated by 132 citations (for example Zhao Y. et al. Nat. Commun. 2019;10(1):768. with ChIP-PCR for mouse cells).

Anti-p300 (Santa Cruz sc-585; western blotting 1:1000). Validated by 450 citations (for example Bell E. et al. Nat. Commun. 2020;11(1):1112 with ChIP for mouse cells).

Anti-BRD4 (BETHYL A301-985A100; western blotting 1:1000). Validated by the manufacturer with western blot and immunohistochemistry for human and mouse, and also validated by 45 citations (for example Liu L. et al. Cell Stem Cell. 2014;15 (5):574-588 with western blot for mouse cells).

Anti-RbBP5 (BETHYL A300-109A; western blotting 1:1000). Validated by the manufacturer with ChIP, western blot and immunohistochemistry for human, and also validated in 102 citations (for example Hoshii T. et al. Cell 2018;172 (5):1007-1021.e17. with western blot for human cells).

Anti-RING1B (Cell Signaling Technology 5694S; western blotting 1:1000). Validated by the manufacturer with ChIP and immunofluorescence for human, and also validated by 42 citations (for example Blackledge N.P. et al. Mol. Cell 2020;77 (4):857-874.e9. with ChIP for mouse cells).

Anti-H3K27me3 (Millipore 07-449; western blotting 1:1000; immunofluorescence 1:200). Validated by the manufacturer with western blot for human and immunocytochemistry for mouse, and also validated by 1615 citations (for example Scarola M. et al. Nat. Commun. 2015;6:7631. with ChIP for mouse cells).

Anti-Histone H3 (Abcam ab1791; western blotting 1:3000). Validated by the manufacturer with western blot and immunofluorescence for human and mouse, ChIP for mouse, and also validated by 3065 citations (for example Li Q. et al. Cell Rep. 2020;30(2):465-480.e6. with western blot and immunoprecipitation for mouse cells).

Anti-PHF20 (Cell Signaling Technology 3934; western blotting 1:1000). Validated by the manufacturer with western blot and immunoflurescence for human, and also validated by 20 citations (for example Klein B.J. et al. Cell Rep. 2016;17(4):1158-1170. with western blot for human cells).

Anti-ACTIN (Sigma A2066; western blotting 1:1000). Validated by the manufacturer with western blot, immunofluorescence and immunohistochemistry for chicken and human, and also validated by 2485 citations (for example Falach R. et al. Sci. Rep. 2020;10(1):9007. with western blot for human cells).

Anti-E-cadherin (BD Biosciences 610181; western blotting 1:1000). Validated by the manufacturer with western blot, immunofluorescence and immunoprecipitation for human, mouse and rat, and also validated by 963 citations (for example Nava M.M. et al. Cell 2020;181(4):800-817.e22. with immunofluorescence for human cells).

Anti-H3K27ac (Abcam ab4729, 5 µg/ChIP). Validated by the manufacturer with ChIP, western blot and immunofluorescence for human, mouse and rat, and also validated by 1150 citations (for example Vaid R. et al. Nucleic Acids Res. 2020;48(9):4877-4890. with ChIP and western blot for Drosophila cells).

Anti-Pol II (Santa Cruz sc-899, 10 µg/ChIP). Validated by the manufacturer with western blot and immunoflurescence for human, mouse and rat, and also validated by 733 citations (for example Bywater M. et al. Nat. Commun. 2020;11(1):1827. with ChIP for mouse cells).

Mouse anti-IgG (Beyotime A7028, 5 or 10 µg/ChIP). Validated by 31 citations (for example Liang S. et al. Autophagy 2019;1-15. with ChIP for mouse cells).

Rabbit anti-IgG (Beyotime A7016, 5 or 10 µg/ChIP). Validated by 37 citations (for example Liu L. et al. Cell Stem Cell 2014;15 (5):574-588. with ChIP for mouse cells).

Goat anti-IgG (Beyotime A7007, 5 or 10 µg/ChIP). Validated by 5 citations (for example Liu L. et al. Cell Stem Cell 2014;15 (5):574-588. with ChIP and immunoprecipitation for mouse cells).

Anti-CD44-APC (eBiosciences 17-0441; flow cytometry 1:200). Validated by the manufacturer with immunofluorescence and flow cytometry for human and mouse, and also validated by 191 citations (for example O'Malley J. et al. Nature 2013;499 (7456):88-91. with flow cytometry for mouse cells).

Anti-ICAM1-PE (eBiosciences 12-0542; flow cytometry 1:200). Validated by the manufacturer with flow cytometry for mouse, and also validated by 9 citations (for example Wang H. et al. Cell Res. 2015;25(6):674-690. with flow cytometry for mouse cells).

Eukaryotic cell lines

Policy information about cell lines

Cell line source(s)

Platinum-E (Plat-E) cells were obtained from The Fourth Military Medical University (Xi'an, China).
HEK293T was purchased from the ATCC.
pre-iPSC #2-2, OG2 ESCs, OSK-iPSCs and OG2 EpiSCs were obtained from Dr. Jiekai Chen (Guangzhou Institutes of Biomedicine and Health, Chinese Academy of Sciences, China).
Rex1GFPd2-ESCs were obtained from Dr. Yangming Wang (Peking University, Beijing, China).
Drosophila S2 cells were obtained from Dr. Jianguo He (Sun Yat-sen University, Guangzhou, China)(purchased from Invitrogen, R69007).

Authentication

OG2 ESCs, OG2 EpiSCs, OSK-iPSCs, pre-iPSCs and Drosophila S2 cells were tested by RT-PCR after lysis with Trizol for specific gene expression.

Mycoplasma contamination

All cell lines were routinely tested to ensure they are mycoplasma negative.

Commonly misidentified lines
(See ICLAC register)

No cell lines used in this study were found in the database of commonly misidentified cell lines that is maintained by ICLAC and NCBI Biosample.

Animals and other organisms

Policy information about studies involving animals; ARRIVE guidelines recommended for reporting animal research

Laboratory animals

E13.5 of the crossed offsprings of 129 female mice (purchased from Beijing Vital River Laboratory Animal Technology Co., Ltd.) and OG2 transgenic male mice (purchased from the Jackson Laboratory) were used to isolate OG2 MEFs.
E13.5 of the crossed offsprings of male/female B6N-Jmjd3tm1Nju mice (purchased from Nanjing Biomedical Research Institute of Nanjing University) and OG2 transgenic female/male mice (purchased from the Jackson Laboratory) were used to isolate Jmjd3 cKO OG2 MEFs.
ICR mouse blastocysts were used to form chimeras through iPSC injection.

Wild animals

This study did not involve wild animals.

Field-collected samples

This study did not involve field-collected samples.

Ethics oversight

Animal experiments in this paper were compliant with all relevant ethical regulations regarding animal research, and were conducted under the approval of the Institutional Animal Care and Use Committee of Guangzhou Institutes of Biomedicine and Health under licence number 2014013.

Note that full information on the approval of the study protocol must also be provided in the manuscript.

ChIP-seq

Data deposition

☒ Confirm that both raw and final processed data have been deposited in a public database such as GEO.

☒ Confirm that you have deposited or provided access to graph files (e.g. BED files) for the called peaks.

Data access links
May remain private before publication.

Sequencing data are available in GEO database under the accession number GSE75005 and GSE137627.

Files in database submission

MEF Jmjd3-/- anti-H3K27me3
MEF Jmjd3-/- input
MEF Jmjd3+/+ anti-H3K27me3
MEF Jmjd3+/+ input
OSKM Day 10 Jmjd3-/- FLAG overexpression anti-H3K27me3
OSKM Day 10 Jmjd3-/- JMJD3 overexpression anti-H3K27me3
OSKM Day 10 Jmjd3+/+ FLAG overexpression anti-H3K27me3
OSKM Day 10 Jmjd3+/+ JMJD3 overexpression anti-H3K27me3
OSKM Day 5 Jmjd3-/- FLAG overexpression anti-H3K27me3
OSKM Day 5 Jmjd3-/- JMJD3 overexpression anti-H3K27me3
OSKM Day 5 Jmjd3+/+ FLAG overexpression anti-H3K27me3
OSKM Day 5 Jmjd3+/+ JMJD3 overexpression anti-H3K27me3
OSKM Day 5 Jmjd3+/+ FLAG overexpression anti-KLF4
OSKM Day 5 Jmjd3+/+ JMJD3 overexpression anti-KlLF4
OSKM Day 5 Jmjd3+/+ FLAG overexpression anti-H3K27ac
OSKM Day 5 Jmjd3+/+ JMJD3 overexpression anti-H3K27ac
OSKM Day 5 FLAG-JMJD3 overexpression anti-FLAG
OSlowKM Day 5 FLAG-JMJD3 overexpression anti-FLAG
OSKM Day 5 FLAG-JMJD3 overexpression input
OSlowKM Day 5 FLAG-JMJD3 overexpression input

Genome browser session
(e.g. UCSC)

Not applicable.

Methodology

Replicates

There are no biological replicates for ChIP-seq in this study.

Sequencing depth

sample_name, #reads, #unique mapped reads, read_length, read_type
k27me3_KO_flag_OSKMd10, 21376723, 13480301, 50, single-end
k27me3_KO_flag_OSKMd5, 21733899, 13778563, 50, single-end
k27me3_KO_j3_OSKMd10, 22588512, 14265832, 50, single-end
k27me3_KO_j3_OSKMd5, 20688511, 12812277, 50, single-end
k27me3_KO_mef, 21263813, 12342652, 50, single-end
k27me3_WT_flag_OSKMd10, 21836619, 14214310, 50, single-end
k27me3_WT_flag_OSKMd5, 24263096, 15373660, 50, single-end
k27me3_WT_j3_OSKMd10, 24475181, 15512067, 50, single-end
k27me3_WT_j3_OSKMd5, 21628322, 13112978, 50, single-end
k27me3_WT_mef, 23127791, 13626252, 50, single-end
k27ac_WT_flag_OSKMd5, 35724935, 30354748, 50, single-end
k27ac_WT_j3OX_OSKMd5, 40226258, 34822969, 50, single-end
klf4_WT_flag_OSKMd5, 38804305, 26074230, 50, single-end
klf4_WT_j3OX_OSKMd5, 44223174, 29563042, 50, single-end
input-KO-MEF_R1, 23331674, 14676676, 50, single-end
input-WT-MEF_R1, 23383617, 14838782, 50, single-end
Mm_oskm_flagjmjd3, 44549467, 28009895, 2x75, paired-end
Mm_oskm_input, 41530414, 24049301, 2x75, paired-end
Mm_osmlowk_flagjmjd3, 51641113, 31921536, 2x75, paired-end
Mm_osmlowk_input, 56286758, 33794278, 2x75, paired-end

Antibodies

The following antibodies were used for ChIP-seq:
anti-H3K27me3 (Millipore 07-449, 5 ug/ChIP)
anti-H3K27ac (Abcam ab4729, 5 ug/ChIP)
anti-KLF4 (R&D AF3158, 10 ug/ChIP)
anti-FLAG (Sigma F1804, 10 ug/ChIP)

Peak calling parameters

bowtie2 -p 4 --very-sensitive --end-to-end --no-unal -U ${inp} -x mm10 -S ${out}.sam

```
macs2 callpeak -g mm  -t klf4_WT_flag_OSKMd5.bam -n klf4_WT_flag_OSKMd5
macs2 callpeak -g mm  -t klf4_WT_j3OX_OSKMd5.bam -n klf4_WT_j3OX_OSKMd5
macs2 callpeak $opts -t Mm_oskm_flagjmjd3.rp1.bam -c Mm_oskm_input.rp1.bam -n Mm_oskm_flagjmjd3
macs2 callpeak $opts -t Mm_osmlowk_flagjmjd3.rp1.bam -c Mm_osmlowk_input.rp1.bam -n Mm_oslowkm_flagjmjd3
samtools view -q 30 -b $f | bedtools bamtobed | grep -v 'chrUn_' | grep -v 'random' | grep -v 'chrM' |awk '{FS=OFS="\t";
print $1,$2,$3,".",0,$6}' - | gzip -9 >$rn
run_dfilter.sh -std=5 -d=k27me3_WT_mef.bed -bs=100 -ks=60 -refine -o=k27_WT_mef.bed
run_dfilter.sh -std=5 -d=k27me3_KO_mef.bed -bs=100 -ks=60 -refine -o=k27_KO_mef.bed
run_dfilter.sh -std=5 -d=k27me3_KO_flag_OSKMd10.bed -bs=100 -ks=60 -refine -o=k27_KO_flag_d10.bed
run_dfilter.sh -std=5 -d=k27me3_KO_j3_OSKMd10.bed -bs=100 -ks=60 -refine -o=k27_KO_j3_d10.bed
run_dfilter.sh -std=5 -d=k27me3_WT_flag_OSKMd10.bed -bs=100 -ks=60 -refine -o=k27_WT_flag_d10.bed
run_dfilter.sh -std=5 -d=k27me3_WT_j3_OSKMd10.bed -bs=100 -ks=60 -refine -o=k27_WT_j3_d10.bed
run_dfilter.sh -std=5 -d=k27me3_KO_flag_OSKMd5.bed -bs=100 -ks=60 -refine -o=k27_KO_flag_d5.bed
run_dfilter.sh -std=5 -d=k27me3_KO_j3_OSKMd5.bed -bs=100 -ks=60 -refine -o=k27_KO_j3_d5.bed
run_dfilter.sh -std=5 -d=k27me3_WT_j3_OSKMd5.bed -bs=100 -ks=60 -refine -o=k27_WT_j3_d5.bed
run_dfilter.sh -std=5 -d=k27me3_WT_flag_OSKMd5.bed -bs=100 -ks=60 -refine -o=k27_WT_flag_d5.bed
```

Data quality

Reads were quality controlled using FASTQC. Reads with a mapping quality score >30 were retained for analysis.
ChIP-seq data quality was judged from the 'predicted fragment length' value produced by MACS2 (v2.2.5). For the DFilter H3K27me3, data was visually inspected and correlated against previously published H3K27me3 ChIP-seq data (where appropriate).

Number of peaks, >5-fold enriched, #peaks FDR<5%, #Total peaks
klf4_WT_flag_OSKMd5, 5700, 10768, 10768
klf4_WT_j3OX_OSKMd5, 6836, 14495, 14495
k27_WT_mef, 6955, 6955, 6955
k27_KO_mef, 4144, 4144, 4144
k27_KO_flag_d10, 5377, 5377
k27_KO_j3_d10, 4879, 4879, 4879
k27_WT_flag_d10, 5132, 5132, 5132
k27_WT_j3_d10, 3046, 3046, 3046
k27_KO_flag_d5, 5065, 5065, 5065
k27_KO_j3_d5, 6540, 6540, 6540
k27_WT_j3_d5, 5056, 5056, 5056
k27_WT_flag_d5, 6429, 6429, 6429
Mm_oskm_flagjmjd3_peaks, 9483, 14068, 14068
Mm_oslowkm_flagjmjd3_peaks, 1148, 2285, 2285

Software

Mouse reads from ChIP-seq data for H3K27me3, H3K27ac, JMJD3, and KLF4 were aligned to the mm10 genome using bowtie2 (v2.4.1), and Drosophila reads for H3K27me3 were aligned to Drosophila dm3 genome.
Peaks were called using DFilter (v1.6) for H3K27me3, and MACS2 (v2.2.5) for KLF4 and JMJD3.
Motif discovery was performed using HOMER (v4.10.3).

Flow Cytometry

Plots

Confirm that:

- [x] The axis labels state the marker and fluorochrome used (e.g. CD4-FITC).
- [x] The axis scales are clearly visible. Include numbers along axes only for bottom left plot of group (a 'group' is an analysis of identical markers).
- [x] All plots are contour plots with outliers or pseudocolor plots.
- [x] A numerical value for number of cells or percentage (with statistics) is provided.

Methodology

Sample preparation

For cell surface marker CD44/ICAM1 analysis, reprogramming cells were digested with 0.05% trypsin (for day 5) or 0.5% type IV collagenase followed with 0.05% trypsin, suspended in flow cytometry buffer (2% FBS in PBS), and stained with antibodies ICAM1-PE (eBiosciences, 12-0542; 1/200) and CD44-APC (eBiosciences, 17-0441; 1/200) at 4°C for 30 minutes avoiding light and followed by washing with flow cytometry buffer for three times, then cells were filtered with a membrane to remove cell clusters.
For the Oct4-GFP/Dppa5a-dTomato dual reporter MEF reprogramming analysis, cells were digested with 0.5% type IV collagenase followed with 0.05% trypsin at the indicated time points and were filtered with a membrane to remove cell clusters.
For OG2 EpiSCs and Rex1GFPd2 EpiLCs reprogramming analysis, cells were digested with accutase at the indicated time points and filtered with a membrane to remove cell clusters.

Instrument

A LSRFortessa (BD Biosciences) machine was used for data collection of CD44/ICAM1 surface marker analysis, and a BD Accuri C6 Plus machine was used for other flow cytometry data collection.

Software	Flow cytometry data were collected with BD FACSDiva software (v8.0.1) or BD Accuri C6 Plus software (1.0.23.1), and analyzed by Flowjo (v10.4).
Cell population abundance	Most of the cells (around 80%) were within the post-sorting fractions in the preliminary FSC/SSC gating.
Gating strategy	We set the preliminary FSC/SSC gates based on cell size and complexity to remove debris and other events of non-interest. For the CD44/ICAM1 surface marker analysis, the gates for positive cells were determined by non staining cells. For flow cytometry analysis of other reporter cell lines, we set the gate for positive populations based on the negative MEF control .

☒ Tick this box to confirm that a figure exemplifying the gating strategy is provided in the Supplementary Information.
