# Supplementary material for: JMJD3 acts in tandem with KLF4 to facilitate reprogramming to pluripotency
Source: Nat Commun. 2020 Oct 8;11:5061. doi: 10.1038/s41467-020-18900-z (PMC7545202; doi:10.1038/s41467-020-18900-z)
Supplement: Supplementary file 4 — Description of Additional Supplementary Files [file 41467_2020_18900_MOESM4_ESM.pdf]

Descriptional of Additional Supplementary Files

File Name: Supplementary Data 1

Description: List of proteins identified by mass spectrometry analysis of JMJD3 and UTX complexes, OSKM+Vc – day 5.

File Name: Supplementary Data 2

Description: List of primers used in this study.

File Name: Supplementary Data 3

Description: Differentially expressed genes from RNA-seq data.

File Name: Supplementary Data 4

Description: Reads of H3K27me3 ChIP-seq; H3K27me3 levels at TSS and TTS of all transcripts, and at ESC-specific enhancers; H3K27me3 and H3K27ac levels at KLF4 peaks.
